# Versatile electrical stimulator for cardiac tissue engineering—Investigation of charge-balanced monophasic and biphasic electrical stimulations

**DOI:** 10.3389/fbioe.2022.1031183

**Published:** 2023-01-04

**Authors:** Stefano Gabetti, Antonio Sileo, Federica Montrone, Giovanni Putame, Alberto L. Audenino, Anna Marsano, Diana Massai

**Affiliations:** ^1^ Department of Mechanical and Aerospace Engineering and PolitoBIOMed Lab, Politecnico di Torino, Turin, Italy; ^2^ Department of Surgery and Department of Biomedicine, University Hospital Basel, University of Basel, Basel, Switzerland

**Keywords:** biomimetic approach, electrical stimulation, monophasic waveform, biphasic waveform, charge balance, cardiac tissue engineering, in vitro models, parallel investigation

## Abstract

The application of biomimetic physical stimuli replicating the *in vivo* dynamic microenvironment is crucial for the *in vitro* development of functional cardiac tissues. In particular, pulsed electrical stimulation (ES) has been shown to improve the functional properties of *in vitro* cultured cardiomyocytes. However, commercially available electrical stimulators are expensive and cumbersome devices while customized solutions often allow limited parameter tunability, constraining the investigation of different ES protocols. The goal of this study was to develop a versatile compact electrical stimulator (ELETTRA) for biomimetic cardiac tissue engineering approaches, designed for delivering controlled parallelizable ES at a competitive cost. ELETTRA is based on an open-source micro-controller running custom software and is combinable with different cell/tissue culture set-ups, allowing simultaneously testing different ES patterns on multiple samples. In particular, customized culture chambers were appositely designed and manufactured for investigating the influence of monophasic and biphasic pulsed ES on cardiac cell monolayers. Finite element analysis was performed for characterizing the spatial distributions of the electrical field and the current density within the culture chamber. Performance tests confirmed the accuracy, compliance, and reliability of the ES parameters delivered by ELETTRA. Biological tests were performed on neonatal rat cardiac cells, electrically stimulated for 4 days, by comparing, for the first time, the monophasic waveform (electric field = 5 V/cm) to biphasic waveforms by matching either the absolute value of the electric field variation (biphasic ES at ±2.5 V/cm) or the total delivered charge (biphasic ES at ±5 V/cm). Findings suggested that monophasic ES at 5 V/cm and, particularly, charge-balanced biphasic ES at ±5 V/cm were effective in enhancing electrical functionality of stimulated cardiac cells and in promoting synchronous contraction.

## 1 Introduction

*In vivo*, cardiac cells reside in a highly dynamic microenvironment and continuously experience pulsed electrical excitation followed by cyclic contraction (Torrent-Guasp et al., 2004; Ethier and Simmons, 2007; Kuznetsova et al., 2008). Such physical conditions are essential for maintaining tissue homeostasis and can be involved in the pathogenesis of several cardiac diseases (Jacot et al., 2010; McCain and Parker, 2011; Lyon et al., 2015; Pesce et al., 2017; Garoffolo et al., 2022). The myocardium is a very complex tissue characterized by an anisotropic helical network of muscle fibers, based on tightly packed rod-shaped cardiomyocytes (CMs) and cardiac fibroblasts (CFs) embedded in a collagen-rich extracellular matrix (ECM) with a dense vasculature (Zhou and Pu, 2016; Buckberg et al., 2018; Litviňuková et al., 2020). Responsible for the heart contraction are the CMs, whose interconnected syncytium allows to respond by synchronized contraction to the cyclic electrical pulses propagating along the cardiac muscle fibers (Monteiro et al., 2017; Montero et al., 2020). In the last two decades, several Cardiac Tissue Engineering (CTE) approaches demonstrated that for producing *in vitro* functional substitutes of myocardial tissue it is fundamental to provide a biomimetic culture environment replicating the most relevant physical stimuli of the myocardium, such as cyclic stretch and/or electrical pulses (Massai et al., 2013; Guilak et al., 2014; Hirt et al., 2014b; Mattei et al., 2014; Vunjak Novakovic et al., 2014; Stoppel et al., 2016; Scuderi and Butcher, 2017; Weinberger et al., 2017; Pitoulis et al., 2020; Stein et al., 2020; Carlos-Oliveira et al., 2021; Zhuang et al., 2022).

To mimic the cyclic movement of the ventricles, several customized bioreactors and commercial devices have been developed and adopted for imposing cyclic stretch to stretchable substrates or three-dimensional (3D) constructs, leading to enhanced cell proliferation, myocardium-like organization of the cultured constructs, and increased contractile performance of engineered cardiac tissues (Eschenhagen et al., 2002; Zimmermann et al., 2002; Birla et al., 2007; Pang et al., 2009; Rubbens et al., 2009; Tulloch et al., 2011; Dhein et al., 2014; Mihic et al., 2014; Massai et al., 2020; Putame et al., 2020; Tran et al., 2021). In parallel, further studies demonstrated that *in vitro* electrical stimulation (ES) affects the rate, duration, and number of action potentials of CMs, increasing the percentage of spontaneously beating cells and promoting cell–cell coupling and calcium handling (Radisic et al., 2004; Sathaye et al., 2006; Nunes et al., 2013; Hernández et al., 2016). ES is commonly delivered to the cultured cardiac cells or tissues as field stimulation, i.e., by the application of an electric field between two parallel electrodes immersed in the culture medium (Tandon et al., 2009; Maidhof et al., 2012; Pavesi et al., 2014; Zhang et al., 2021). In literature, two main pulsed ES modes have been proposed and tested: monophasic and biphasic ES waveforms. Pioneering studies used monophasic ES, which is simple to generate, and demonstrated an improvement in the cardiac cell electrical coupling, an increase in the production of the functional Connexin 43 (Cx-43), and an enhancement in the cell inter-connectivity (Radisic et al., 2004; Tandon et al., 2011). Biphasic ES was firstly considered as an alternative ES mode for reducing the accumulation of by-products in the culture medium resulting by faradaic reactions at the electrode-medium interfaces (Tandon et al., 2009). Preliminary comparative studies showed that biphasic ES induced higher levels of maturation in neonatal rat CMs (Chiu et al., 2011) and in human cardiac progenitor cells (Pietronave et al., 2014) compared to monophasic ES (Lasher et al., 2012; Hirt et al., 2014a; Visone et al., 2018a; Visone et al., 2018b; Zhang et al., 2021; Pretorius et al., 2022). However, apart from the two mentioned studies, a clear advantage of biphasic ES was not reported in literature and monophasic ES has been widely adopted in CTE to promote cell maturation (Ronaldson-Bouchard et al., 2018; Zhao et al., 2019).

To deliver monophasic or biphasic pulsed ES modes in CTE applications, different setups have been developed, mostly connecting the electrodes to commercial electrical stimulators (Tandon et al., 2011; Maidhof et al., 2012; Chan et al., 2013; Xiao et al., 2014; Baumgartner et al., 2015; Ruan et al., 2016; Kroll et al., 2017; LaBarge et al., 2019; Ronaldson-Bouchard et al., 2019; Zhao et al., 2019) or with pacemakers (Martherus et al., 2010). However, commercial electrical stimulators are expensive and cumbersome devices and often allow limited modulation of ES parameters, constraining the investigation of different ES conditions and ultimately hindering the adoption of ES protocols in CTE (Ronaldson-Bouchard et al., 2019). In recent years, the availability of affordable open-source micro-controllers promoted the development of customized stimulators to implement ES for biomimetic CTE approaches (Pavesi et al., 2014; Ronaldson-Bouchard et al., 2019; Béland et al., 2020). However, these platforms are affected by limitations such as low ES tunability, reduced adaptability to different cell culture setups, and often limited versatility in terms of ES mode.

Inspired by this scenario, we developed a versatile compact electrical stimulator (ELETTRA) designed for delivering parallelizable pulsed ES for CTE applications in a stable, accurate, and controlled way and at a competitive cost. Open-source and low-cost technologies were adopted for the stimulator development. To test the ELETTRA performances, customized culture chambers were designed and manufactured. Computational modelling supported the characterization of the spatial distribution of the electric field and the current density within the culture chamber. Accuracy and compliance tests were performed to characterize the ES parameters delivered by ELETTRA and its reliability during cell culture. Lastly, to investigate the influence of different ES modes on the *in vitro* maturation of cardiac cells, neonatal rat cardiac cells were exposed to monophasic or biphasic pulsed ES delivered by ELETTRA. The biological effects of the different applied ES modes were evaluated in terms of electrical functionality, cardiomyocyte contractility, percentages of areas positive for Cx-43 and Sarcomeric α-actinin, and sarcomeric organization.

## 2 Materials and methods

### 2.1 ELETTRA electrical stimulator

The following design requirements guided the development of the ELETTRA electrical stimulator. Firstly, it was designed for providing *in vitro* electrical stimuli in the range of the physiological pulsatile electric field experienced by human cardiac cells *in vivo* (electric field = 0.1–10.0 V/cm, resting rate = 1.0–1.7 Hz, pulse duration = 1–2 ms (Tandon et al., 2009; Klingensmith, 2020)). Moreover, inspired by several studies (Chiu et al., 2011; Tandon et al., 2011; Maidhof et al., 2012; Pietronave et al., 2014; Ronaldson-Bouchard et al., 2018; Visone et al., 2018a), ELETTRA was developed to deliver a voltage-controlled stimulation either as monophasic or as biphasic square wave pulses. With a view to reduce the number of sequential experiments and to increase the number of possible conditions to be tested simultaneously, parallelization, modularity, and versatility guided the stimulator development. Lastly, ELETTRA was devised to be compact and easy to use in a cell culture laboratory following conventional Good Laboratory Practices (GLP). During the development process, an iterative optimization approach, together with low-cost manufacturing and assembly procedures, allowed progressively refining the ELETTRA features.

**FIGURE 1:**
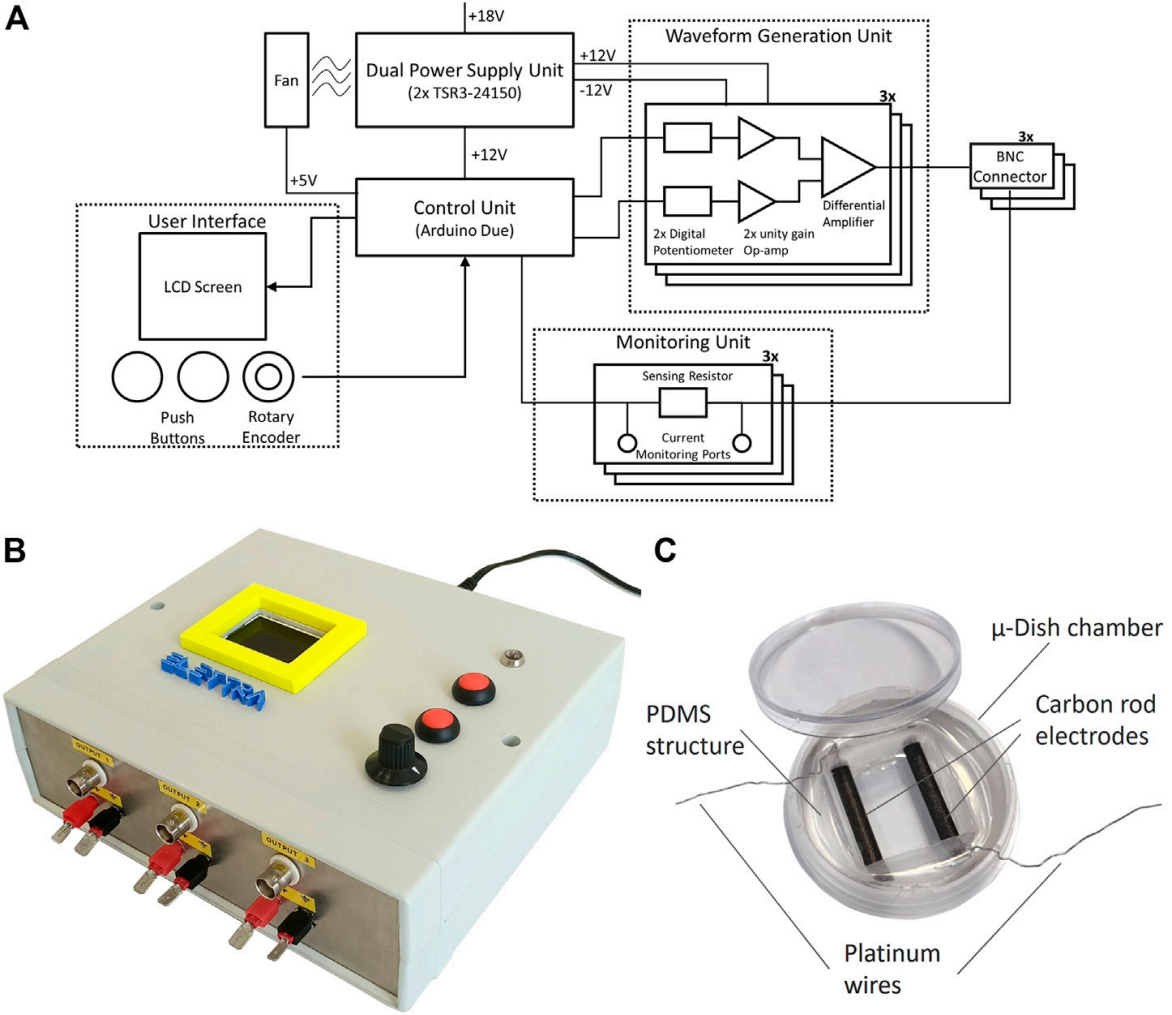
ELETTRA and culture chamber. **(A)** Schematic drawing of ELETTRA showing the relations between its subsystems and components. **(B)** Picture of the ELETTRA electrical stimulator. **(C)** Picture of the assembled culture chamber.

In detail, ELETTRA is embedded in a compact case (21 cm × 18 cm x 7 cm) and it is composed of five subsystems: 1) the control unit; 2) the waveform generation unit; 3) the dual power supply unit; 4) the monitoring unit; and 5) the user interface (Figure 1A). The control unit consists of an Arduino Due micro-controller board (Arduino, Italy) running a purpose–built software. The code, loaded on the micro-controller, communicates with the user interface and allows controlling multiple outputs with accurate timing. A USB port allows direct connection to the micro-controller board for possible software update, without disassembling the device. The waveform generation unit enables generating square-wave stimuli with monophasic or biphasic waveform, controllable in pulse duration (1–10 ms), voltage (0.25–12 V), and frequency (0.5–10 Hz), with a maximum output current of 700 mA, for three independent outputs in view of stimulating multiple constructs in parallel (Table 1). For each output, a circuit based on two digital potentiometers (MCP41010, Microchip Technologies, United States), two unity gain amplifiers (LM358, STMicroelectronics, Italy) and a differential amplifier (L272M, On Semiconductor, United States), converts the digital signals from two Arduino Due pins to the stimulation voltage waveform. The dual power supply unit, based on two DC/DC converters (TSR-3 24150, TracoPower, Switzerland), regulates the voltage from a standard AC/DC adapter to ±12 V dual supply, enabling the delivery of biphasic stimulations. A fan guarantees the system cooling. The electric circuits were assembled on RoHS-compliant printed circuit boards (PCBs), designed by using the open-source free software KiCAD 5.1.6 and externally manufactured (JLCPCB, China). The monitoring unit is based, for each output, on a sensing resistor (10 Ω) placed downstream of the reference electrode port and two standard connectors mounted on the stimulator frame. During stimulation, by connecting an oscilloscope to the connectors and measuring the voltage drop on the sensing resistor for each output, the user can indirectly measure the current flowing between the electrodes. Finally, the user interface is composed of a LCD display, two push buttons, and a rotary encoder that allow setting the stimulation parameters for each independent output and switching on/off the device. During the stimulation, the elapsed time and the stimulation parameters are showed. ELETTRA can be connected to different experimental set-ups by Bayonet Neill–Concelman (BNC) sockets (Figure 1B). For the here adopted set-up, an output cable equipped with a splicing connector (Wago, Germany) enabled connecting up to six culture chambers in parallel.

**TABLE 1:** ELETTRA stimulation parameters.

Parameter	Range
Waveform type	Monophasic/Biphasic
Voltage (V)	0.25–12.00
Frequency (Hz)	0.5–10.0
Pulse duration (ms)	1–10

### 2.2 Culture chambers

In view of investigating the influence of different ES modes on the *in vitro* maturation of cardiac cells, 16 culture chambers to be connected to ELETTRA were developed (see Supplementary Material S1). Each culture chamber is based on a μ-Dish chamber (diameter = 35 mm; ibidi GmbH, Germany), in which a polydimethylsiloxane (PDMS, Sylgard 184, Dow Corning, United States) cylindrical structure with a central rectangular hole is press-fit inserted. Two carbon rod electrodes (length = 26 mm, diameter = 3 mm; Sigma-Aldrich, Germany), linked to platinum wires (diameter = 0.3 mm; Polyfil AG, Switzerland) to be connected to ELETTRA, are embedded in parallel within the PDMS structure at a fixed distance of 1 cm, with their facing sides exposed to air/culture medium for a length of 20 mm (Figure 1C). Carbon rods were selected among different electrode materials due to their properties of biocompatibility, charge transfer, and resistance to corrosion (Tandon et al., 2006). The PDMS structure is autoclavable and, once sterilized, it can be press-fitted in a sterile μ-Dish chamber. Each culture chamber can house up to 2.5 ml of culture medium.

### 2.3 ELETTRA characterization

#### 2.3.1 Lumped-parameter model

For characterizing the voltage and the current waveforms provided by ELETTRA, an equivalent lumped-parameter model of the culture chamber and of the ELETTRA waveform generation unit was developed and simulations were run (Simulink, MathWorks, United States). The culture chamber was modelled as a Simplified Randles Cell circuit (Randles, 1947; Tandon et al., 2009; Chang and Park, 2010; Khademi and Barz, 2020), which is composed of three key elements: 1) the resistor *R*_*e*_ (resistance of the solution); 2) the resistor *R*_*p*_ (electrodes’ resistance to corrosion); and 3) the capacitor *C*_*p*_ (non-ideal double layer at the electrode/electrolyte interface), with *R*_*p*_ and *C*_*p*_ put in parallel. Considering the materials and the geometry of the manufactured culture chambers, the following values were adopted: 1) *R*_*e*_ = 53 Ω; 2) *R*_*p*_ = 5.13 × 10^8^ MΩ; 3) *C*_*p*_ = 240 µF (for details on adopted assumptions see Supplementary Material S1).

The ELETTRA waveform unit was modelled by its ideal equivalent circuit. The simulations were run varying the number of chambers (from 1 to 6) connected in parallel to ELETTRA and applying a monophasic waveform (voltage = 5 V, frequency = 1 Hz, pulse duration = 2 ms).

#### 2.3.2 Electric field finite element analysis

For characterizing the spatial distribution of the electric field and of the current density within the culture chamber, a finite element analysis (FEA) was performed (Comsol Multiphysics 5.3, Comsol Inc., United States). The geometry of the culture chamber, composed of five sub-domains (PDMS structure; carbon rod electrodes; culture medium; polyethylene derivate coverslip and chamber; air above the culture medium), was meshed with 1.7 × 10^6^ tetrahedral elements and 1.7 × 10^5^ triangular elements. Each sub-domain was assumed as a homogenous isotropic medium with specific electrical properties (Supplementary Table S1). For the culture medium, 2.5 ml were modelled, and the cell monolayer was assumed as part of the culture medium volume due to its high water content (Pavesi et al., 2014). Using the Electric Currents interface in the AC/DC module, a stationary simulation was performed, solving the continuity equation in absence of distributed current sources:

$$\nabla ∙J=\nabla ∙\left(\sigma E+J_{e}\right)=0$$

where *J* is the current density (A/m^2^), *σ* is the electrical conductivity (S/m), *E* is the electric field distribution (V/m), and *Je* is the externally generated current density (A/m^2^), which was set to 0 in the simulation. The electric field distribution was then derived by computing the gradient of the electric potential *V*:

E=−∇V

As boundary conditions, uniform electric potentials at the external sides of the electrodes (5 V at the positive electrode, 0 V at the ground electrode) and electric insulation at the external faces of the model were imposed.

#### 2.3.3 Experimental in-house tests

The ELETTRA performances in terms of compliance of delivered stimulation parameters and reliability during cell culture application were preliminary tested in-house. A culture chamber was filled with 2.5 ml of culture medium (Dulbecco’s Modified Eagle Medium (DMEM), Sigma-Aldrich, United States) and was connected to ELETTRA. Each of the three outputs was set to deliver monophasic ES or biphasic ES (frequency = 1 Hz, pulse duration = 2 ms) varying the voltage from 1 to 12 V with steps of 1 V (from ±1 V to ±12 V for the biphasic ES). By using a digital oscilloscope (PicoScope 2204A, Pico Technologies, United Kindom) and connecting the probe to the positive output and to the ground port of the monitoring unit on ELETTRA’s chassis, the delivered voltage was measured. A second oscilloscope probe was connected to the monitoring ports of ELETTRA to measure the voltage drop across the sensing resistor. For both measurements, 10 consecutive pulses were recorded. Recorded values were compared with the nominal voltage value set on ELETTRA. The percentage errors of measured values with respect to nominal values were calculated as the mean of the differences between the imposed voltage and the measured voltage, and were expressed as mean ± standard deviation (SD). The current waveforms were extracted by dividing the voltage drop on the sensing resistor by its resistance value. For each test, the current peak was extracted and its mean and SD were calculated. Subsequently, the reliability of ELETTRA in stimulating multiple culture chambers in parallel was tested. From 1 and up to six culture chambers were filled with 2.5 ml of DMEM and were connected in parallel to ELETTRA. Monophasic and biphasic stimulations (voltage = 5 V for monophasic mode, ±5 V for biphasic mode; frequency = 1 Hz; pulse duration = 2 ms) were delivered. Tests were repeated for each output, the current was monitored connecting the oscilloscope probe to the ELETTRA monitoring ports, 10 consecutive pulses were recorded and the mean and SD of the current peak were calculated. For each characterization test, voltage waveforms were recorded at a sample rate of 780 kS/s. Acquired signals were post-processed using MATLAB R2020b (MathWorks, United States). Experimental results for monophasic stimulation were then compared with the lumped parameter model outcomes. Moreover, data from the current measurements were used for setting the voltage for biological tests, in order to deliver the desired electric field to the cell culture for each tested condition.

### 2.4 Cell culture experiments

#### 2.4.1 Cell isolation

Neonatal rat cardiac cells (CMs and CFs) were isolated from 2-3-days old Sprague Dawley rats as previously described (Radisic et al., 2008), according to the Swiss Federal guidelines for animal welfare, and all procedures were approved by the Veterinary Office of the Canton Basel (Basel, Switzerland). Briefly, rat ventricles were cut into small pieces and digested overnight in 0.06% w/v trypsin solution (trypsin from bovine pancreas, Sigma-Aldrich, United States) at 4°C with continuous shaking at 50–60 oscillations per minute. Five continuous 4-min cycles of 0.1% w/v collagenase solution (type 2 collagenase, Worthington-Biochem, United States) treatment were used to continue digestion of the minced tissues. To allow CFs attachment and enrich the cell population for CMs, isolated cardiac cells were pre-plated in culture flasks for 45 min at 37°C and 5% CO_2_. The enriched cardiac population was seeded at a density of 6 × 10^4^ cells/cm^2^ and cultured for 48 h, before starting the experiments, in a seeding medium composed of high glucose DMEM (Sigma-Aldrich, United States), supplemented with 1% Hepes buffer (Sigma-Aldrich, United States), 1% Penicillin/Streptomycin (Sigma-Aldrich, United States), 1% l-glutamine (Sigma-Aldrich, United States) and 10% Fetal Bovine Serum (FBS, Sigma-Aldrich, United States).

#### 2.4.2 Cell characterization

After isolation, cardiac cells were harvested, washed with phosphate buffered saline (PBS, Sigma-Aldrich, United States), fixed for 20 min at 4°C [4% paraformaldehyde (PFA, Sigma-Aldrich, United States)], permeabilized for 15 min at room temperature [0.5% Triton 100X (Sigma-Aldrich, United States) in PBS] and stained for 30 min at 4°C with the following antibodies: anti-Sarcomeric α-actinin antibody [conjugated with phycoerythrin (PE), Miltenyi Biotec, Germany] and anti-cardiac Troponin T antibody (conjugated with allophycocyanin (APC), Miltenyi Biotec, Germany). All the antibodies were used at 1:200 dilution in FACS buffer (PBS w/o Ca^2+^ and Mg^2+^ (Sigma-Aldrich, United States), 0.5% v/v FBS (Sigma-Aldrich, United States) and 2 mM ethylenediaminetetracetic acid [EDTA (Sigma-Aldrich, United States)]. Life and dead staining was performed using violet fluorescent reactive dye [read at BV421 (Invitrogen by Thermo Scientific, United States) diluted 1 µl in 1 ml PBS (Sigma-Aldrich, United States)]. Cells were then suspended in FACS buffer and at least 50.000 events per sample were collected with a flow cytometer (LSRfortessa, BD, United States).

#### 2.4.3 2D cell culture under ES

**FIGURE 2:**
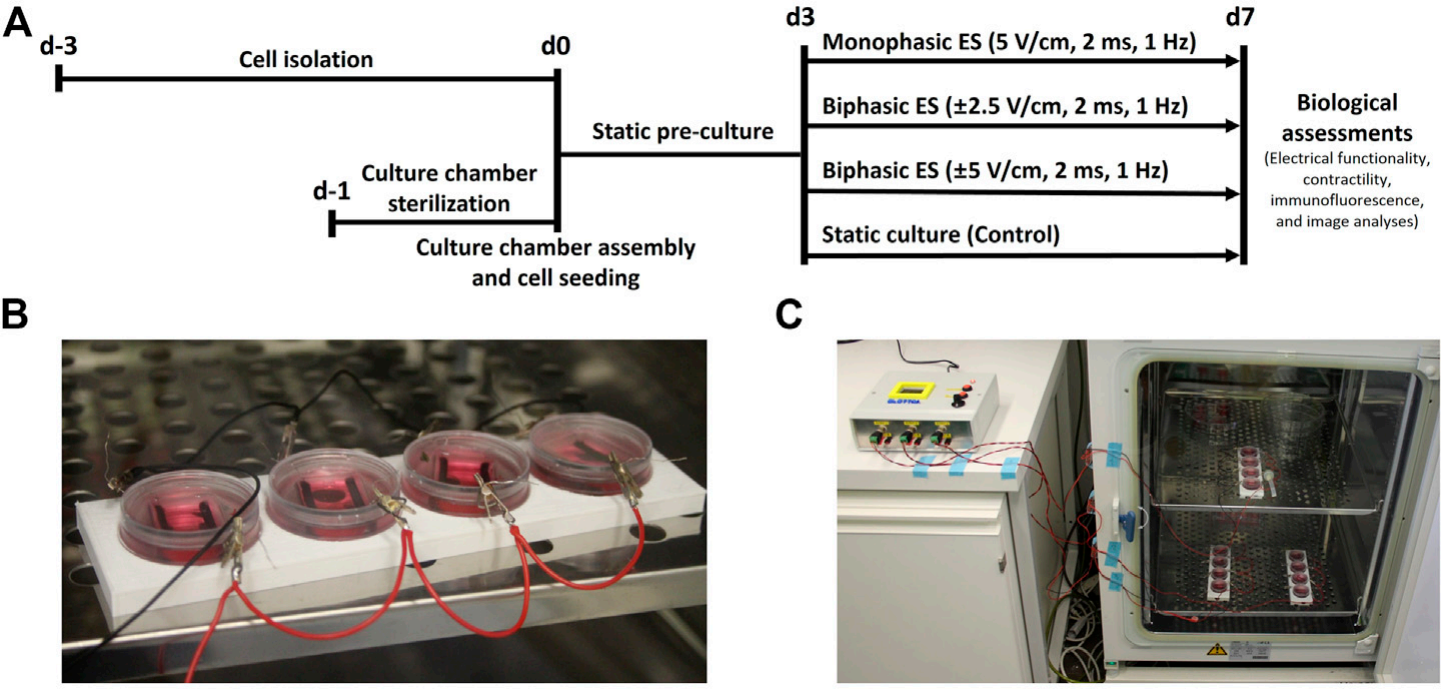
Biological experiments. **(A)** Timeline of the performed culture protocols. **(B)** Picture of four culture chambers connected in parallel during the experiments. **(C)** Picture of the whole setup during the biological experiments: each output of ELETTRA is connected to a set of four culture chambers placed inside the incubator.

Before starting the experiments, the autoclaved PDMS structures were press-fit inserted in the µ-Dish chambers. Cells were then seeded at a density of 6 × 10^4^ cells/cm^2^ corresponding to 2.5 × 10^5^ cells per chamber using 2.5 ml of seeding medium. From the following day, the seeding medium was changed with 2.5 ml of low glucose DMEM (Sigma-Aldrich, United States), supplemented with 1% Hepes buffer (Sigma-Aldrich, United States), 1% Penicillin/Streptomycin (Sigma-Aldrich, United States), 1% l-glutamine (Sigma-Aldrich, United States), and 1% FBS (Sigma-Aldrich, United States) to limit CFs proliferation (culture medium). For each condition, from three to four samples were statically pre-cultured for 3 days, to allow the cell recovery from the isolation process. A 3-day pre-culture step, without ES, was chosen to let neonatal rat cardiac cells recover from the isolation from heart tissue as previously described (Radisic et al., 2004; Tandon et al., 2009; Hirt et al., 2014a). Cardiac cells were then cultured for additional 4 days without (control) or with ES, in order to evaluate the effects of the ES in the short term as in previous publications (Tandon et al., 2008; Chiu et al., 2011; Pavesi et al., 2014; Pietronave et al., 2014). Culture medium was changed every 2 days to provide fresh nutrients to the cells and to remove toxic by-products that may be produced during the ES. Three different ES modes were tested simultaneously (frequency = 1 Hz, pulse duration = 2 ms): 1) monophasic ES at 5 V/cm; 2) biphasic ES at ± 2.5 V/cm; 3) biphasic ES at ± 5 V/cm (Figure 2A). The biphasic ES at ± 2.5 V/cm mode was chosen to deliver the same absolute value of the electric field variation of the monophasic ES at 5 V/cm mode, while the biphasic ES at ± 5 V/cm mode was chosen to deliver the same amount of charge released by the monophasic ES at 5 V/cm mode (Supplementary Figure S1). ES modes were applied to three or four culture chambers in parallel (Figure 2B), and all samples were cultured in total for 7 days in a standard incubator (37°C, 95% humidity, 5% CO_2_) (Figure 2C). Two independent experiments were conducted, obtaining at least six replicates for each condition.

#### 2.4.4 Electrical functionality and cardiomyocyte contractility assessments

After 7 days of culture, cell contractile activity in response to external electrical pacing was assessed by measuring two electrical maturation parameters, the Excitation Threshold (ET) and the Maximum Capture Rate (MCR) (Marsano et al., 2016). The pacing tests were performed inside a live-imaging microscope incubator (ZEISS X91, Olympus, Japan), with controlled temperature (37°C) and CO_2_ (5%). Electrical pulses (1 Hz, 2 ms) were imposed using ELETTRA and, starting from 1 V/cm, a progressively increasing voltage was applied to determine the minimum electric field needed for generating a synchronous cell contraction (ET). Once ET was established, the electric field was imposed equal to 150% ET and, increasing the frequency, the maximum frequency that the cells can follow (MCR) was assessed. Movies of the electrically paced cells were acquired using ×10 objective lens at 30 fps with the live-imaging microscope incubator. Data from functionality assessment were analysed by GraphPad Prism 8 (GraphPad Software, United States) adopting Kruskal–Wallis test with *post hoc* Dunn’s multiple comparison test. Data were averaged and expressed as the mean ± SD. The statistical significance was denoted as ∗ for *p*-value ≤0.05, ∗∗ for *p*-value ≤0.01, and ∗∗∗ for *p*-value ≤0.001 (*n* ≥ 6 replicates for each condition, from two independent experiments). Moreover, from the recorded movies of the electrically paced cells, CM contractility was assessed by measuring the peak amplitude (PA) of the contractions, defined as the maximum displacement of each CM during a contraction, and the contraction time delay (CTD), defined as the maximum time delay between the contractions of different CMs following a single pacing pulse (Supplementary Figure S4E). For each culture condition, the movies recorded when imposing an external electrical pacing with electric field equal to the ET value and frequency equal to 1 Hz were considered. The movies were analyzed with TrackMate, a Fiji software (NIH, United States) tracking plugin, and processed with a custom Matlab code (see more details in Supplementary Material S1).

#### 2.4.5 Immunofluorescence and image analysis

To investigate the cardiac maturation and the CMs/CFs ratio at the end of the culture, immunofluorescence analysis was performed. Cells were washed with PBS (Sigma-Aldrich, United States) and fixed using 4% PFA (Sigma-Aldrich, United States) for 15 min. Afterwards, the immunostaining was carried out. In detail, cells were washed 2 times with PBS (Sigma-Aldrich, United States), then they were incubated for 1 h at room temperature in 5% normal goat serum (Sigma-Aldrich, United States) with 0.25% Triton 100X (Sigma-Aldrich, United States) in PBS (Sigma-Aldrich, United States). After washing 2 times with PBS, cells were incubated for 1 h in the dark with the following primary antibodies: mouse monoclonal anti-Sarcomeric α-actinin (ABCAM9465, Abcam, United Kingdom) and rabbit polyclonal IgG anti-Connexin-43 (C6219, Sigma-Aldrich, United States). Cells were again washed twice with PBS and then incubated in the dark for 30 min with fluorescently labelled Alexa546 anti-mouse and Alexa647 anti-rabbit secondary antibodies (Life Technologies, Thermo Fisher Scientific, United States). Nuclei were stained using 4′,6-diamidino-2-phenylindole (DAPI, Invitrogen, Thermo-Fisher Scientific, United States) at 1:40 for 15 min. Incubations were performed at room temperature and antibodies were diluted in PBS 1X with 0.1% bovine serum albumin (BSA, Sigma-Aldrich, United States). Primary and secondary antibody dilution was 1:200. 10 immunofluorescence staining images of four samples for each condition, from two independent experiments, were acquired using ×40 objective lenses on a Nikon-CSU1 spinning-disk confocal microscope (Nikon, Japan), and subsequently analyzed using Fiji. The number of cells was assessed counting the DAPI positive nuclei, while the numbers of CMs and CFs were defined counting the number of cells positive and negative for the Sarcomeric α-actinin, respectively. All the acquired images were then processed with the threshold technique “Intermodes” to segment Cx-43-positive entities and the threshold technique ‘‘Li dark method’’ to segment Sarcomeric α-actinin-positive entities. With these techniques, the percentage of area positive for Cx-43 and the percentage of area positive for Sarcomeric α-actinin were quantified (Navaei et al., 2017; Lemme et al., 2018; Isu et al., 2020; Pisanu et al., 2022). Finally, to understand the portion of CMs which underwent a good maturation in terms of sarcomeric organization, the percentage of CMs with sarcomeric organization with respect to the total number of CMs was quantified (Mytsyk et al., 2019).

## 3 Results

### 3.1 ELETTRA characterization

#### 3.1.1 Experimental in-house tests and lumped parameter model

**FIGURE 3:**
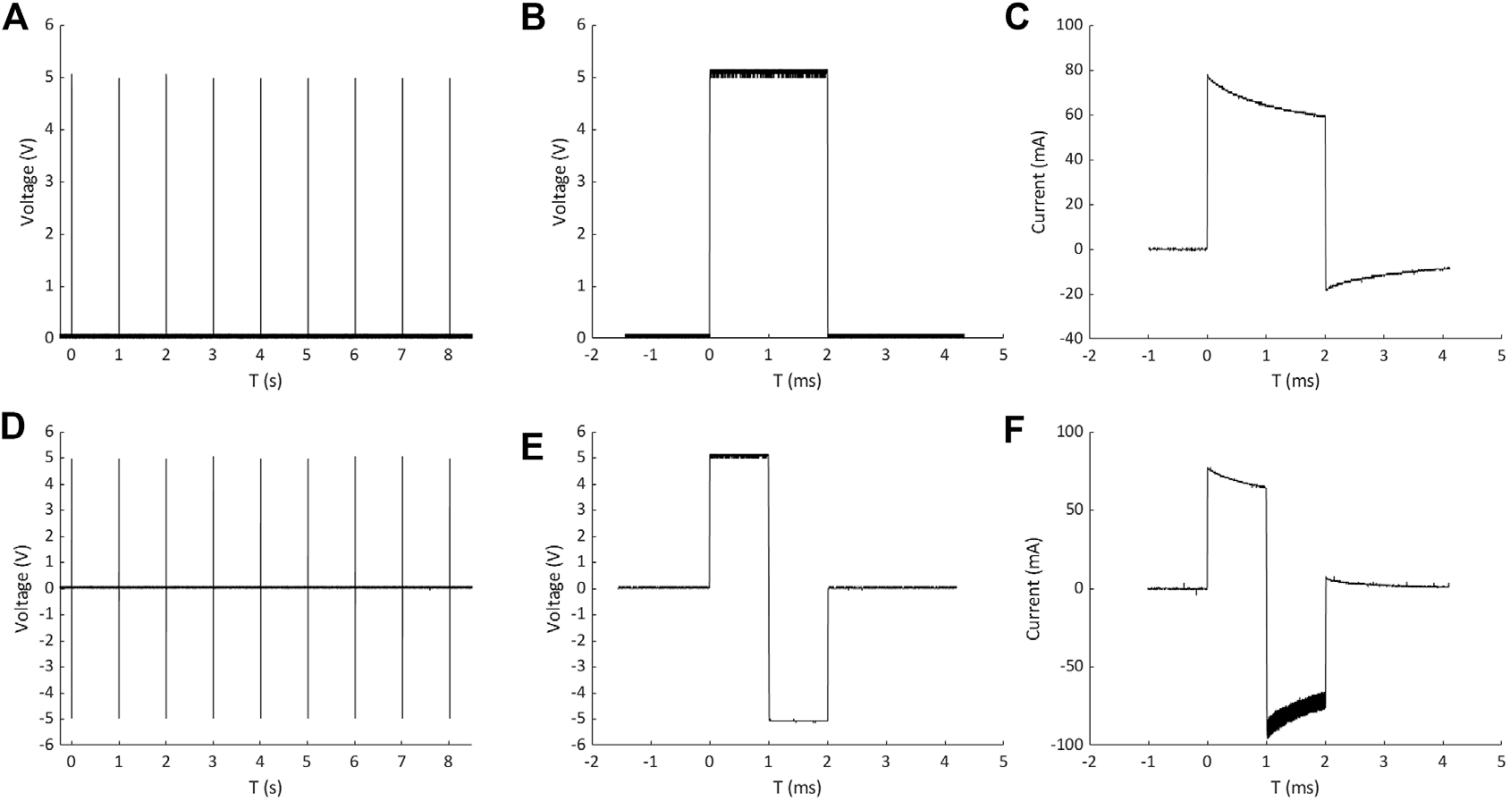
Measurements of pulse train, voltage waveform and resulting current waveform on a single culture chamber for monophasic ES **(A,B and C)** and biphasic ES **(D,E and F)**.

ELETTRA performances were first characterized experimentally. The measurements of the pulse train, the voltage waveform, and the resulting current waveform on a single culture chamber filled with culture medium are shown in Figure 3 for a monophasic (5 V, 1 Hz, 2 ms, Figures 3A–C) and for a biphasic (±5 V, 1 Hz, 2 ms, Figures 3D–F) stimulation, respectively. The pulse train graphs demonstrate that, with respect to the imposed frequency, ELETTRA delivered the stimulations accurately (Figures 3A,D). The voltage waveform graphs show square waves with low noise (noise root mean square voltage = 0.022 V for both ES modes, Figures 3B,E). The percentage errors of the measured voltage with respect to the imposed one were lower than 5% for imposed voltages between 2 and 11 V, with a maximum error of 8.1% at 1 V for monophasic ES (Supplementary Figure S2). As regards the current flowing between the electrodes (Figures 3C,F), for both stimulations the current increased instantly in magnitude when ELETTRA was switched on, while during the active phases the current magnitude decreased due to the induced polarization of the culture medium and the consequent shielding effect of charges accumulated at the electrode-solution interfaces. During the passive phases, the current reversed its direction as the accumulated charges were released in the solution. Moreover, for the biphasic ES, the greater negative peak in current amplitude was due to the combination of the release of charges accumulated during the positive half-wave with the current directly induced by the applied negative voltage (Figure 3F). For both waveform modes, different voltages (1–12 V with 1 V step) were imposed and the peak current values measured on each output showed negligible differences among the three outputs (see Supplementary Table S2). The highest peak current value was equal to 170.79 ± 0.56 mA for monophasic ES and equal to 205.80 ± 1.38 mA for biphasic ES, when imposing a 12 V stimulation voltage. Such values are considerably lower than the maximum current output of the stimulator (700 mA), confirming the suitability of ELETTRA to stimulate multiple chambers in parallel.

**FIGURE 4:**
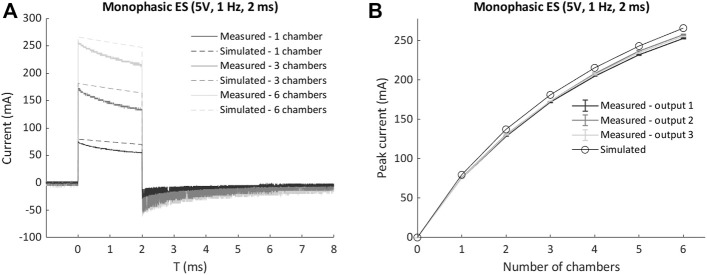
Comparison of measured currents and lumped parameter model results for multiple chambers connected in parallel to ELETTRA. **(A)** Current waveforms for 1, 3, and six chambers connected. **(B)** Peak currents for 1 to six chambers connected.

Up to six culture chambers were then connected in parallel and stimulated with monophasic waveform (5V), and the measured current waveforms and peak currents were compared with the corresponding lumped parameter model results. For each condition, the simulated current was slightly higher than the measured one (Figure 4A), with a maximum discrepancy of the current peak equal to 13 mA (5%) when six chambers were connected in parallel (Figure 4B), because of the lump parameter model neglects the non-ideal behavior of the components and the additional sources of voltage drop (e.g., the connectors). Moreover, a non-linear increase of the peak current occurred for both measured and simulated tests when multiple chambers were connected (Figure 4B). This is related to the voltage drop on the ELETTRA sensing resistor, which increases with the number of chambers connected and generates a consequent decrease of the delivered electric field. Such effect was taken into account during the biological tests, and the voltage was appositely adjusted to deliver the desired electric field to the cell culture for each tested condition.

Concerning the ELETTRA technical specifications as compared to commercially available electrical stimulators usually adopted to deliver ES in CTE applications (i.e., Grass s88x and IonOptix CPace EM), ELETTRA has parallel independent outputs, similarly to the considered commercial devices, and provides comparable peak stimulation current (700 mA) and selectable ranges of voltage (monophasic waveform: 0.25–12 V; biphasic waveform: ±0.25 - ± 12 V) and pulse duration (1–10 ms). Differently, ELETTRA is characterized by a small footprint (21 cm × 18 cm x 7 cm), low weight (0.7 kg), and low cost (€ 300) (for more details see Supplementary Table S3 in Supplementary Material).

#### 3.1.2 Electric field finite element analysis

**FIGURE 5:**
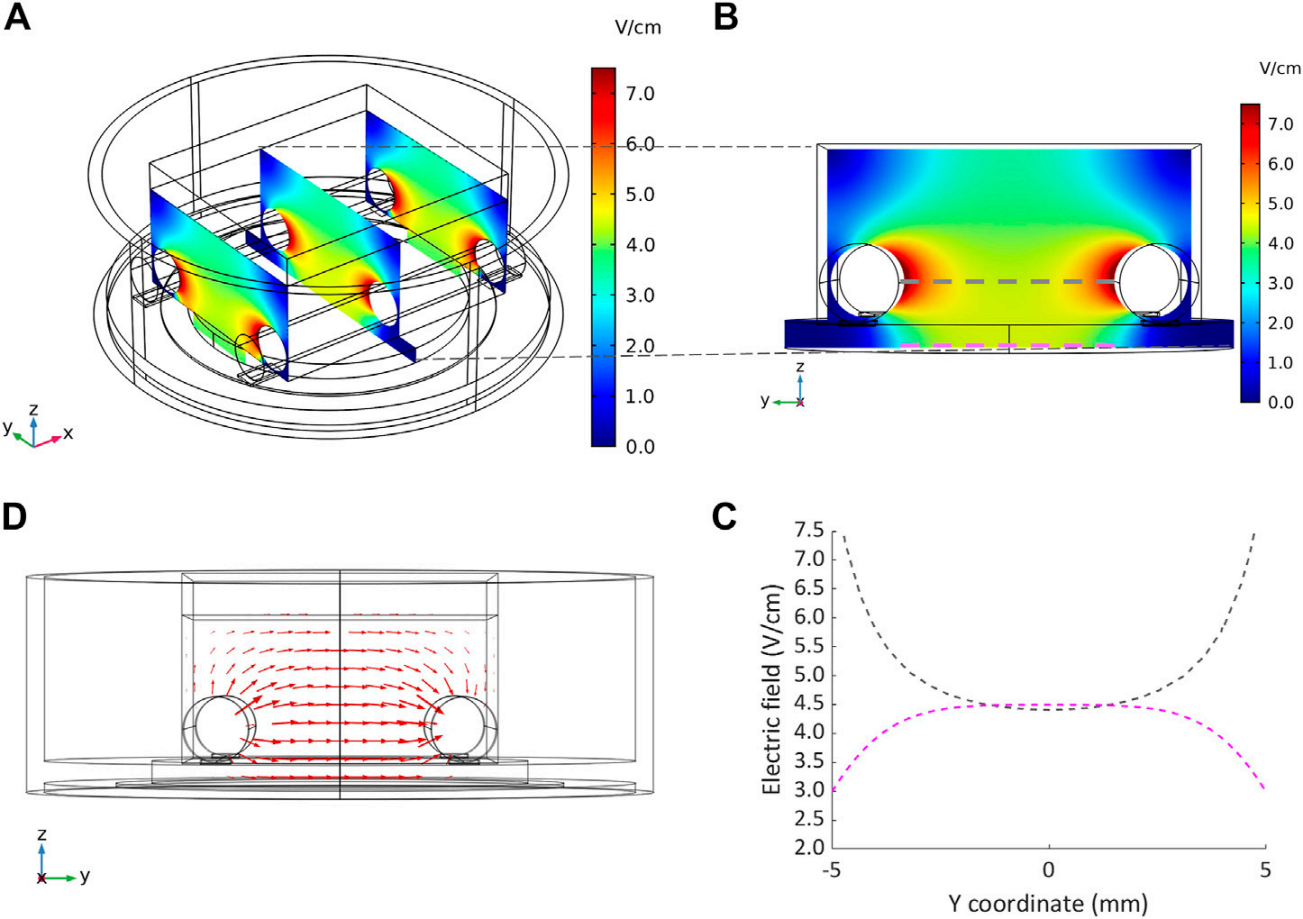
Distributions of the electric field and the current density within the culture chamber. **(A)** Contour plot of electric field magnitude at three planes perpendicular to the electrodes’ main axes, located at x = −10 mm, x = 0 mm, x = 10 mm. **(B and C)** Electric field magnitude on the central plane of the chamber along a line at the height of the electrodes centers and at the bottom of the chamber, respectively. **(D)** Vector diagram of the current density over the central cross section of the culture chamber.

The distributions of the electric field and of the current density within the culture chamber were characterized performing the FEA. Figure 5A shows representative results of the electric field distribution within the culture chamber at three planes perpendicular to the electrodes, when a 5 V voltage and 2.5 ml of culture medium were simulated. The electric field magnitude was about 7.5 V/cm around the electrodes with an almost uniform value (4.5–5 V/cm) between the electrodes (Figure 5A). On the central plane, the electric field magnitude was also calculated along two lines located, respectively, at the height of the electrode centers (gray line, Figure 5B) and at the bottom of the culture chamber where the cells are seeded (magenta line, Figure 5B). At the culture chamber bottom, the electric field magnitude was characterized by an average value of 4.5 V/cm over a wide central region (6 mm) (Figure 5C), while at the height of the electrode centers, it ranged from 4.5 to 7.5 V/cm with a central region (4 mm) characterized by an average value of 4.5 V/cm (Figure 5C).

The vector diagram of the current density over the central cross section of the culture chamber highlights that the direction of the current was well aligned along the electrode-electrode direction, where the cells are cultured (Figure 5D). Due to the uniformity of the electric field, the current density was uniform with an absolute value of 66 mA/cm^2^ in the central region. The total current flowing between the electrodes, calculated as the surface integration of the current density on one electrode, was 112.5 mA, in agreement with the peak current (114.5 mA) measured when a monophasic electrical stimulation (5 V/cm, 2 ms, 1 Hz) was applied to a culture chamber filled with 2.5 ml of culture medium.

### 3.2 Cell culture experiments

#### 3.2.1 Electrical functionality and cardiomyocyte contractility assessments

**FIGURE 6:**
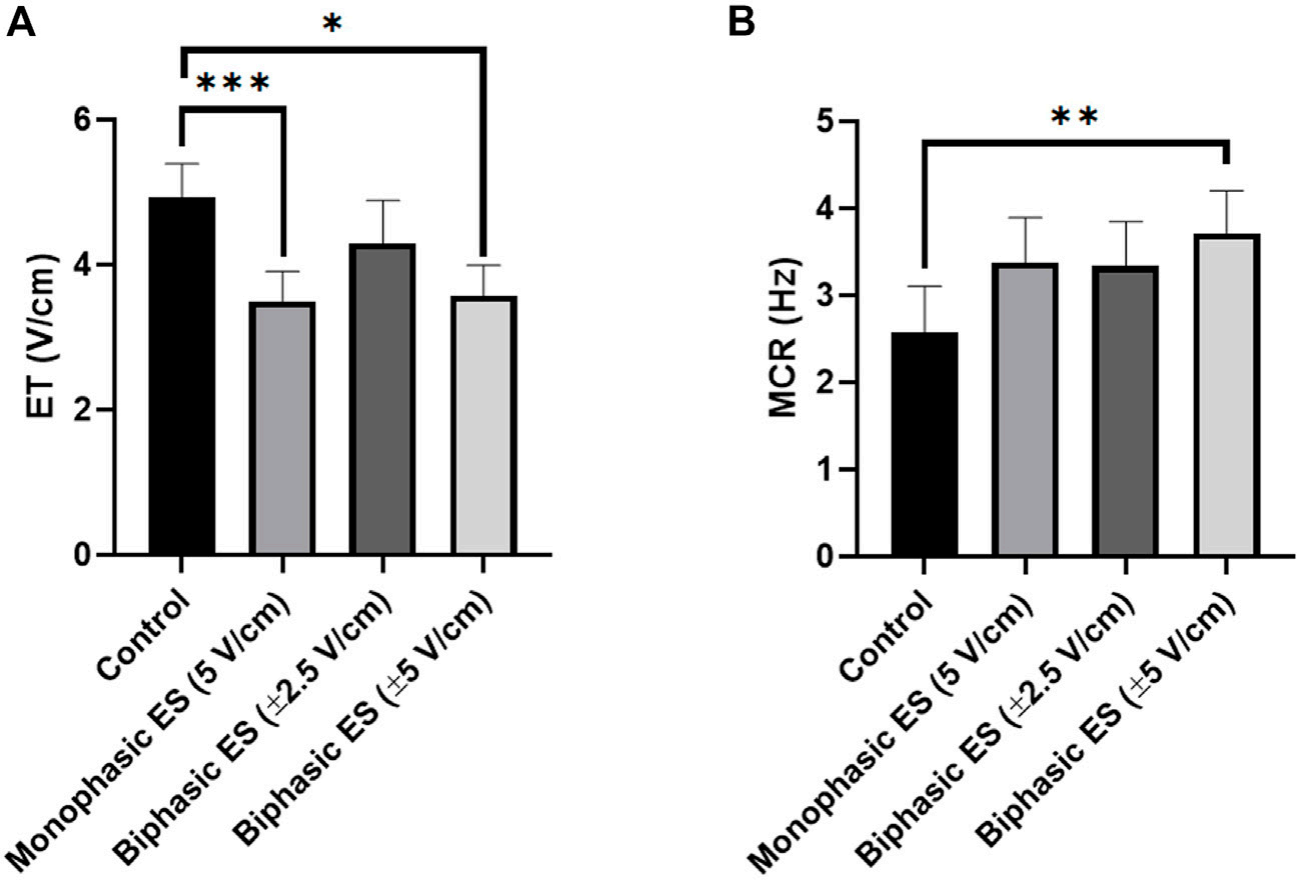
Electrical functionality. **(A)** Excitation threshold (ET) and **(B)** Maximum capture rate (MCR) of CMs for the different culture conditions: Control (no stimulation; n. of replicates = 7); Monophasic ES (5V/cm, 1 Hz, 2 ms; n. of replicates = 8); Biphasic ES (±2.5V/cm, 1 Hz, 2 ms; n. of replicates = 6); Biphasic ES (±5V/cm, 1 Hz, 2 ms; n. of replicates = 7). Results from two independent experiments. Asterisks (*) denote statistically significant difference (*p < 0.05, **p < 0.01, ***p < 0.001).

**FIGURE 7:**
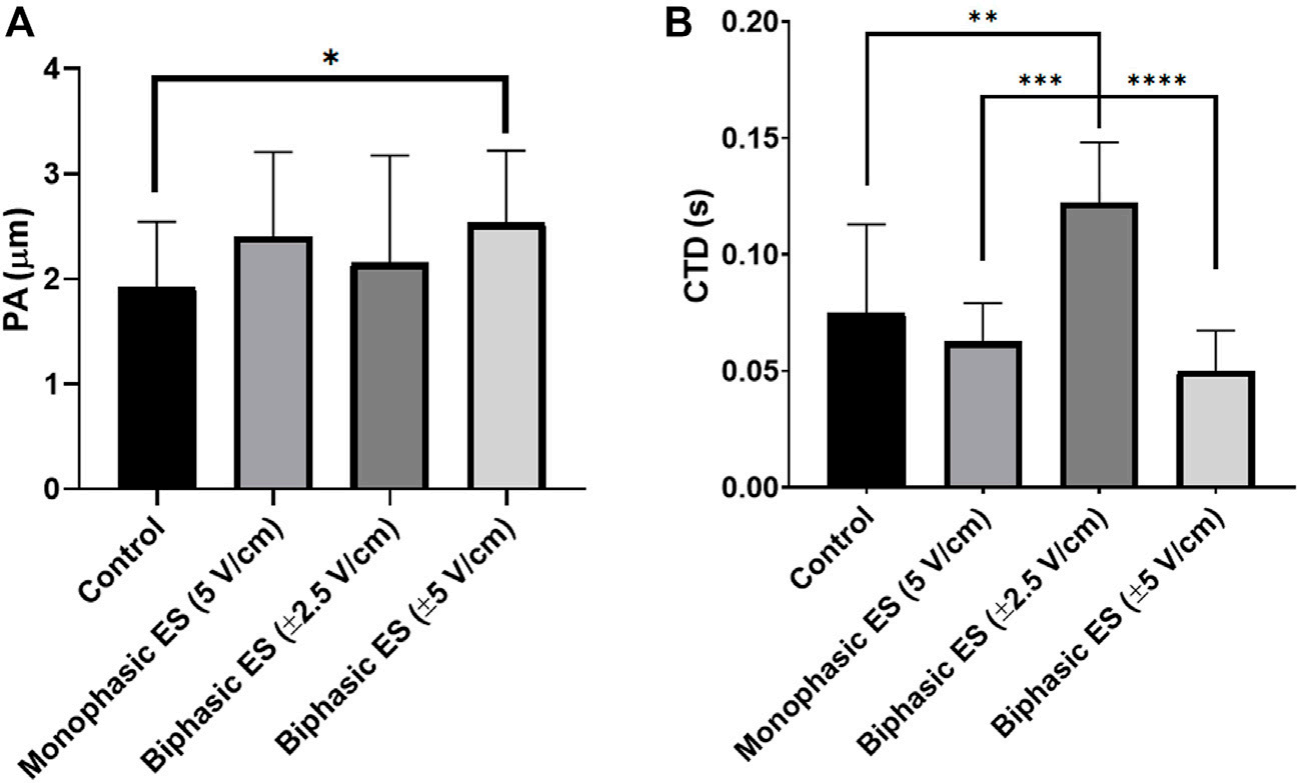
Cardiomyocyte contractility. **(A)** Peak amplitude (PA) and **(B)** contraction time delay (CTD) of CMs for the different culture conditions: Control (no stimulation; n. of replicates = 3); Monophasic ES (5V/cm, 1 Hz, 2 ms; n. of replicates = 4); Biphasic ES (±2.5V/cm, 1 Hz, 2 ms; n. of replicates = 3); Biphasic ES (±5V/cm, 1 Hz, 2 ms; *n*. of replicates = 3). Asterisks (*) denote statistically significant differences (*p < 0.05, **p < 0.01, ***p < 0.001, ****p < 0.0001).

Following 7 days of culture in either static (control) or ES conditions, the electrical functionality of neonatal rat CMs was assessed evaluating their response to an external electrical pacing. Figure 6A shows that, when exposed to external pacing, CMs cultured under monophasic ES at 5 V/cm or under charge-balanced biphasic ES at ±5 V/cm started to synchronously contract at a significantly lower ET (3.50 ± 0.41 V/cm and 3.58 ± 0.41 V/cm, respectively) compared to the control (4.93 ± 0.46 V/cm), but no statistically significant differences were observed among the ES groups. As regards the maximum electrical pacing frequency that the cells can follow, all the ES groups showed an overall increasing trend of MCR compared to the control (2.57 ± 0.53 Hz), however only the CMs cultured under biphasic ES at ±5 V/cm showed a significantly higher MCR (3.71 ± 0.49 Hz). All the ES group reached the frequency around 3 Hz (Figure 6B). The beating frequency of contracting CMs cultured under the different conditions were then expressed as beating rate in beats per minute (bpm) (Supplementary Figure S3). To assess CM contractility, the PA and the CTD were evaluated through the movies acquired during the electrical functionality analysis. Higher PA values characterized all ES conditions compared to control (Figure 7A), indicating the positive effect of the ES, with a remarkable significant difference between biphasic ES at ± 5 V/cm and the control (Figure 7A). Representative graphs of the displacement magnitude of consecutive contractions for the different experimental conditions are reported in Supplementary Material (Supplementary Figure S4). The CTD analysis showed more timed contractions (i.e., lower CTD) of the CMs when cultured with monophasic ES at 5 V/cm and biphasic ES at ±5 V/cm. The biphasic ES at ±2.5 V/cm was significantly different from the other culture conditions (Figure 7B).

#### 3.2.2 Cardiac cell characterization and immunofluorescence analysis

The percentages of CMs and CFs were evaluated both immediately after isolation and at the end of the ES culture. Following the isolation process, cardiac cells were analyzed by flow cytometry and, among the living cells, the CMs resulted to be 67.5 ± 2.9% (double positive for anti-cardiac Troponin T and anti-Sarcomeric α-actinin), while CFs were estimated to be 32.6 ± 2.9% (double negative for the same antibodies). Representative flow cytometry plots are showed in Supplementary Figure S5.

At the end of the culture and after the electrical functionality analysis, the different cultured groups were fixed and stained, and the immunofluorescence image analysis showed almost 55% CMs and 45% CFs for all groups (Supplementary Figure S6).

**FIGURE 8:**
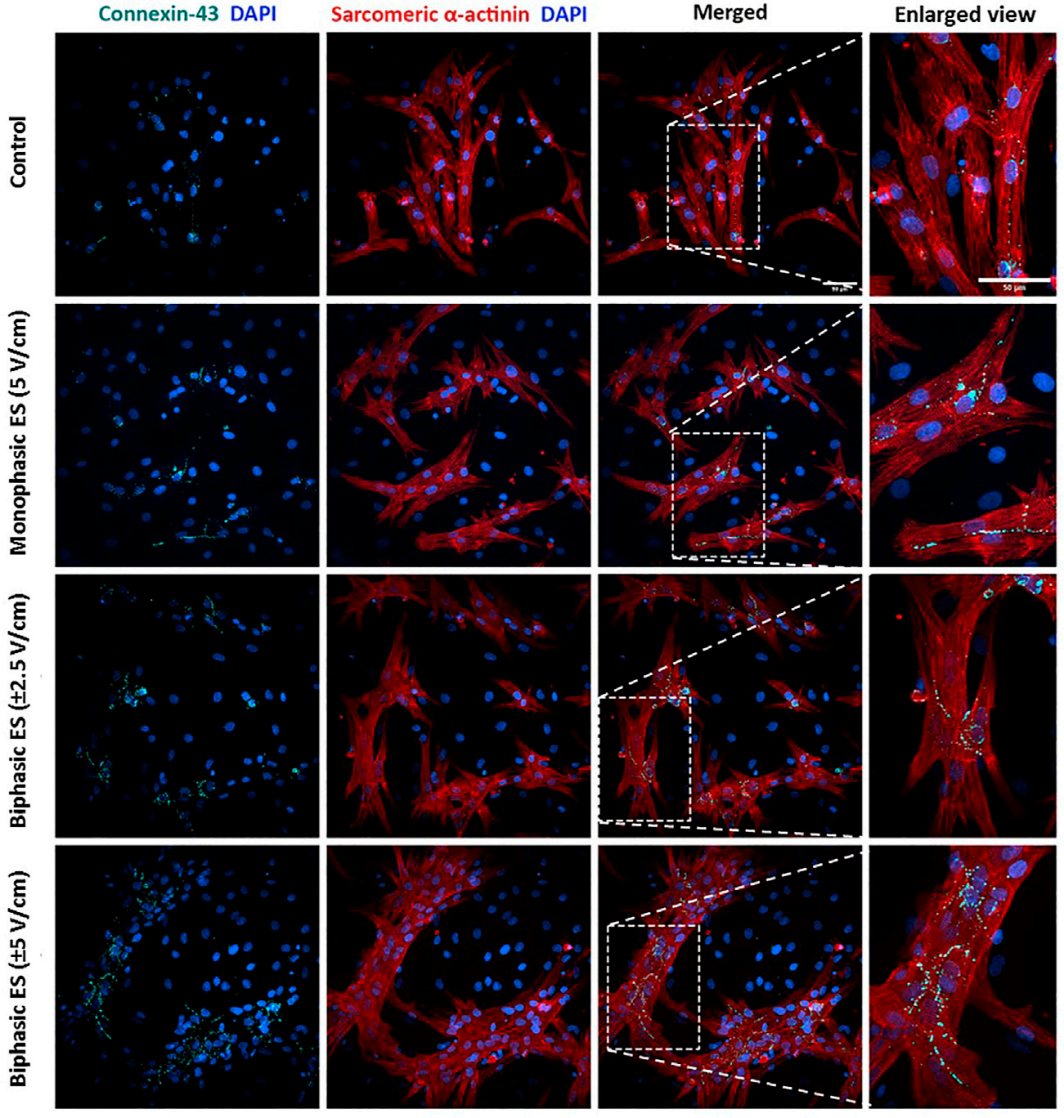
Immunofluorescence images of neonatal rat CMs for the different culture conditions. The images show the Connexin-43 (cyan) with the nuclei in blue (DAPI), Sarcomeric α-actinin (red) with the nuclei in blue (DAPI) in separated images and the merged signals for each experimental group. Scale bar = 50 µm.

**FIGURE 9:**
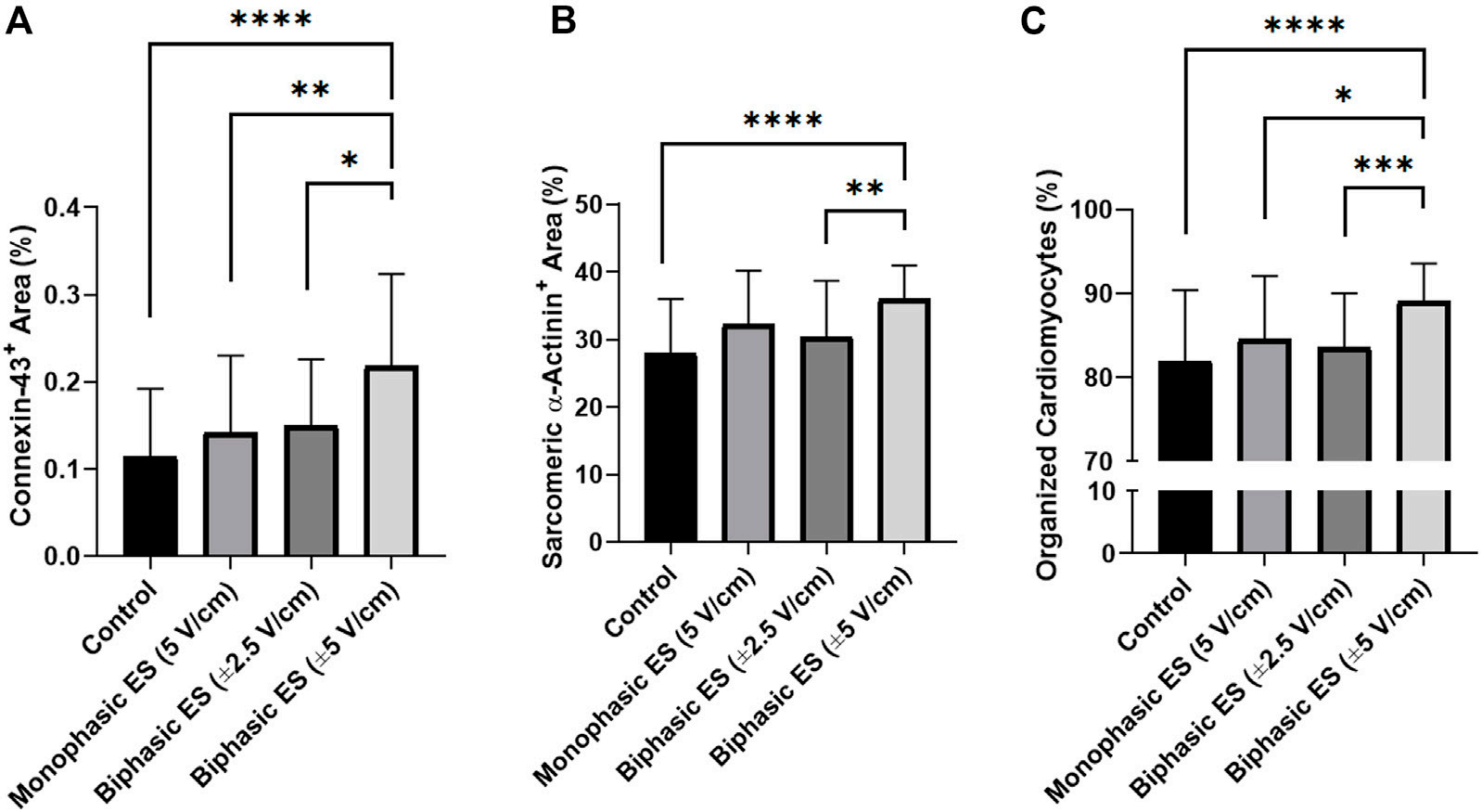
Image-based quantification. **(A)** Percentage of area positive for Connexin-43, **(B)** percentage of area positive for Sarcomeric α-actinin, and **(C)** percentage of organized cardiomyocytes for the different culture conditions: Control (no stimulation; n. of replicates = 4); Monophasic ES (5V/cm, 1 Hz, 2 ms; n. of replicates = 4); Biphasic ES (±2.5V/cm, 1 Hz, 2 ms; *n*. of replicates = 4); Biphasic ES (±5V/cm, 1 Hz, 2 ms; n. of replicates = 4). Results from two independent experiments. Asterisks (*) denote statistically significant differences (*p < 0.05, **p < 0.01, ***p < 0.001, ****p < 0.0001).

To assess the level of cardiac maturation, the presence of specific cardiac proteins, namely the gap-junction protein Cx-43 and the Sarcomeric α-actinin for the contractile structure, was investigated. Unlike the control group, most of the cells cultured under ES were positive for Cx-43 (Figure 8). In particular, cells cultured under monophasic ES at 5 V/cm or under biphasic ES at ±5 V/cm were characterized by the Cx-43 mainly localized at the cell membrane in proximity of neighboring CMs (Figure 8), suggesting its functional role as gap junction. In addition, the experimental group biphasic ES at ±5 V/cm showed significant higher percentages of areas positive for Cx-43 (Figure 9A) and for Sarcomeric α-actinin (Figure 9B) compared to the other culture conditions. For cells cultured under biphasic ES at ±2.5 V/cm, the Cx-43 was present both in the cytoplasm and at the cell membrane. For the control condition, Cx-43 was less present and mainly localized within the cytoplasm (Figure 8). Moreover, electrically stimulated CMs appeared to be characterized by a better organization of sarcomeres compared to control CMs. In particular, CMs cultured under biphasic ES at ±5 V/cm were significantly more organized, in terms of sarcomeres, compared to the other experiment groups (Figure 9C).

## 4 Discussion

In CTE research, it has been demonstrated that for the *in vitro* development of functional cardiac substitutes a controlled culture environment and biomimetic mechanical and/or electrical stimuli are fundamental (Stoppel et al., 2016; Scuderi and Butcher, 2017). As concerns the ES, several studies investigated the effects of pulsed electrical stimuli on cardiac cells and constructs under controlled conditions and their potential in promoting cardiac maturation. In particular, it was shown that *in vitro* pulsed ES affects the rate, duration, and number of action potentials of CMs, improves the organization of sarcomeres and the establishment of gap junctions promoting cell–cell coupling and calcium handling, thereby, increasing the electrical and contractile functionality of stimulated cells (Radisic et al., 2004; Sathaye et al., 2006; Zhang et al., 2021). In most of the studies, pulsed ES was delivered as electric field stimulation by applying a voltage between two parallel electrodes immersed in the culture medium (Tandon et al., 2009; Maidhof et al., 2012), and the electrical stimulus was generated by using commercial electrical stimulators (Tandon et al., 2011; Maidhof et al., 2012; Chan et al., 2013; Xiao et al., 2014; Baumgartner et al., 2015; Ruan et al., 2016; Kroll et al., 2017; LaBarge et al., 2019; Ronaldson-Bouchard et al., 2019; Zhao et al., 2019; Zhang et al., 2021). Although commercial stimulators allow testing different ES patterns simultaneously, they are characterized by limited waveform modulation, high cost, and bulkiness, which limit the adoption of ES protocols in CTE. Alternatively, cost-effective and portable platforms have been purposely developed (Pavesi et al., 2014; Ronaldson-Bouchard et al., 2019; Béland et al., 2020), however such devices are limited to a single output with limited ES tunability and/or can be used only with a specific setup.

Taking into account the limitations of commercial and customized electrical stimulators, we developed the electrical stimulator ELETTRA, appositely designed for biomimetic CTE approaches. Particular attention was paid to guarantee versatility, indeed, ELETTRA can deliver a wide range of biomimetic electrical stimuli (voltage = 0.25–12.0 V, frequency = 0.5–10.0 Hz, pulse duration = 1–10 ms, tunable waveforms) and, being equipped with three standard outputs, it can be coupled to multiple custom-made culture chambers or to already existing bioreactors. Moreover, during the development phase, the choice of the open-source Arduino platform guaranteed high customizability and cost-effectiveness compared to commercial devices and computer-based systems, and the use of custom-designed PCBs avoided cumbersome and heavy parts, leading to a compact and portable device (Figure 1B, Supplementary Table S3). Lastly, the integrated user-friendly interface allows quick setting and easy tuning of the stimulation parameters and provides real-time feedbacks to the operators, facilitating laboratory operations. In order to test the ELETTRA performances in a representative CTE application, we then developed customized culture chambers to be connected to ELETTRA for investigating in parallel the influence of different ES waveforms on the *in vitro* maturation of cardiac cells. Autoclavable PDMS structures, each one including two electrodes and fitting to standard cell culture dishes, were manufactured to be connected in parallel to ELETTRA, allowing ease of use and rapid assembling/disassembling procedures.

Firstly, characterization tests were performed on ELETTRA, without and with culture chambers connected, for assessing reliability and accuracy of the device. As regards the voltage tests (1–12 V), the mean percentage errors between the measured and the nominal voltage values were almost negligible (<5%) for imposed voltages ranging from 2 to 11 V, and did not overcome 9% when 1 V or 12 V were set (Supplementary Figure S2). The higher inaccuracy at 1 V has to be ascribed to the limited resolution of the waveform generation unit, while at 12 V it is related to the output voltage saturating the differential amplifier. However, it should be noted that electric field amplitudes commonly applied in CTE range between 2 and 5 V/cm (Tandon et al., 2011; Stoppel et al., 2016), values that can be accurately delivered by ELETTRA. As concerns the characterization of the current flowing between the electrodes, the tests confirmed the suitability of ELETTRA to stimulate multiple culture chambers in parallel for each output (up to six for an imposed voltage of 5V). Indeed, the lumped parameter model outcomes (for which the culture chambers were modeled as Randles cells) were in good agreement with the experimental results, although they slightly overestimated (∼+5%) the current flowing within the culture chambers (Figure 4). This is related to the fact that the simulation did not account for the non-ideal behavior of the adopted components and neglected possible voltage drops (e.g., at the connectors). Moreover, observing the differences between simulated and experimental data, they were lower at the peak current than during the subsequent active phase (Figure 4A), suggesting that the model was accurate in determining the resistive effects of the solution, while it slightly overestimated the capacitive ones. Thus, as the critical parameter is the peak current, the lumped parameter model can be considered a suitable tool for defining the number of culture chambers that can be connected in parallel to each ELETTRA output and, in case different culture chambers would be used, it can be adapted by appropriately tuning the Randles cell parameters. In addition, the characterization of the current allowed assessing the effect of the sensing resistor and thereby adjusting the imposed voltage during the biological tests for ensuring the desired electric field. Lastly, the FEA model confirmed that the cells seeded at the bottom of the culture chamber, between the electrodes, are exposed to almost uniform electric field and current density, with some perturbations around the electrodes (Figure 5). Furthermore, the simulation results confirmed the suitability of the culture chambers in providing uniform electric field to 3D constructs as well, as the electric field is uniform in magnitude and the current is aligned along the electrode–electrode direction even over an appreciable region at the height of the electrode centers (Figures 5B,C).

Experimental measurements validated the computational results, with a difference in the total current between the simulation and the experimental measurements lower than 2%. For the computational analysis, a stationary condition was adopted thus the transient behavior of the delivered pulses was neglected, however, in accordance with previous studies, such assumption was considered acceptable as the modelled system was much smaller than the wavelengths of interest (Voldman, 2006; Maidhof et al., 2012). Thus, the characterization tests confirmed that ELETTRA is a reliable device in providing accurate and repeatable pulsed ES within a range of interest for CTE applications.

ELETTRA was then adopted for investigating the effect of different ES waveforms on cardiac cell monolayers*.* In detail, cardiac cells isolated from neonatal rats were seeded in the manufactured culture chambers, statically pre-cultured for 3 days and then exposed for 4 days to different ES modes (monophasic ES at 5 V/cm; biphasic ES at ±2.5 V/cm; biphasic ES at ±5 V/cm) provided by ELETTRA. Before starting the experiments, the percentages of CMs and CFs were assessed to be 67.5 ± 2.9% and 32.6 ± 2.9%, respectively. Since after 7 days of culture in either control or ES conditions the percentage of CMs (around 55%) and CFs (around 45%) was similar for all experimental groups, we can conclude that the number of CM was not affected by the ES conditions (Supplementary Figure S6), and the increase of the CF percentage was most likely due to CF proliferation. As regards the imposed ES waveforms, to our knowledge this is the first time that the effects of different biphasic waveforms, equivalent to the monophasic waveform (5V/cm) either in the absolute value of the electric field variation (biphasic ES at ±2.5 V/cm) or in charge (biphasic ES at ±5 V/cm), were compared. Indeed, most of the CTE studies applying ES compared monophasic waveforms with biphasic waveforms balancing the absolute value of the electric field variation, avoiding to investigate the charge balance (Chiu et al., 2011; Pavesi et al., 2014; Pietronave et al., 2014). In our study, the electrical functionality tests revealed that CMs cultured under monophasic ES at 5 V/cm or under charge-balanced biphasic ES at ±5 V/cm showed a significantly lower ET compared to the control, and moreover the CMs cultured under biphasic ES at ±5 V/cm presented a significantly higher MCR. Differently, among the ES groups, the CMs exposed to biphasic ES at ±2.5 V/cm were characterized by low functional properties in terms of both ET and MCR (Figure 6). Movie analysis of CM contractility and image-based quantification of indicators of cardiac maturation (i.e., Cx-43, Sarcomeric α-actinin, and sarcomeric organization) showed positive effects of ES. In particular, the experimental group biphasic ES at ±5 V/cm, with the same charge delivered by the monophasic ES at 5 V/cm, showed higher PA and lower CTD (Figure 7), indicating that this ES mode promoted synchronous contractions. In addition, biphasic ES at ±5 V/cm showed more organized and significantly higher expression of the cell gap-junction and contractile unit proteins compared to the other conditions (Figure 9). The charge delivered by the biphasic ES at ±2.5 V/cm was half of the charge delivered by the other applied ES conditions, thus the cells were overall less stimulated with respect to the other ES groups and presented limited electrical functionality and cell contractility. Our results differ from a previous work (Chiu et al., 2011), where a significant reduction of ET for cells cultured under biphasic ES at ± 2.5 V/cm (frequency = 1 Hz, pulse width = 2 ms) compared to the control was shown, without significant differences compared to monophasic ES at 5 V/cm (frequency = 1 Hz, pulse width = 2 ms). However, in the work of Chiu and colleagues, organoids resembling cardiac myofibers cultivated in matrigel-coated microchannels were used, thus the differences in construct and culture conditions could explain the different outcomes of the two works. In terms of maintenance of cell survival and Cx-43 localization, our findings are in agreement with previous studies (Chiu et al., 2011; Pavesi et al., 2014; Pietronave et al., 2014). Indeed, unstimulated cells showed less Cx-43, mainly localized at the cytoplasm level, while in ES groups Cx-43 was detected particularly at the cell membrane, in proximity of neighboring cells (Figure 8).

Although CFs are known to support electrical propagation through gap junctions (e.g., Cx-43) and to communicate with CMs (Kartha, 2021), the expression of Cx-43 was not evident in the CFs populations based on immunofluorescence staining. Additional investigation of the role of CFs during the electrical stimulation would have been out of the scope of this study.

In conclusion, we developed, characterized, and tested a compact, easy-to-use, versatile electrical stimulator, ELETTRA, appositely developed for biomimetic CTE approaches. The adoption of a customizable micro-controller combined with free and open-source software allowed developing a device offering control accuracy, indirect monitoring, versatility, and portability at a competitive cost (Supplementary Table S3). ELETTRA is combinable with different cell/tissue culture set-ups and allows both testing different stimulation patterns simultaneously and stimulating multiple samples in parallel, representing a powerful tool for CTE investigations. The developed culture chambers ensure delivering uniform electrical stimulation to either cell monolayers or 3D cardiac constructs. The biological experiments, showing for the first time an amplitude- or charge-based comparison between biphasic and monophasic waveforms, demonstrated that monophasic ES at 5 V/cm and particularly charge-balanced biphasic ES at ±5 V/cm were effective in enhancing cardiac electrical functionality and in promoting synchronous contraction.

These findings constitute the basis for the future use of ELETTRA in advanced investigations aimed to identify the effects of different ES protocols and to define the combinations of ES parameters inducing specific biological effects. Moreover, coupled with existing bioreactors, ELETTRA could be used to provide combined biomimetic physical stimuli in a physiologically relevant way, for future production of functional CTE constructs to be used in basic and pre-clinical cardiac research, or ultimately as implantable therapeutic strategies.

## Data Availability

The raw data supporting the conclusion of this article will be made available by the authors, without undue reservation.
