# Supplementary material for: Versatile electrical stimulator for cardiac tissue engineering—Investigation of charge-balanced monophasic and biphasic electrical stimulations
Source: Front Bioeng Biotechnol. 2023 Jan 4;10:1031183. doi: 10.3389/fbioe.2022.1031183 (PMC9846083; doi:10.3389/fbioe.2022.1031183)
Supplement: Supplementary file 1 [file DataSheet1.pdf]

Supplementary Material

Culture chamber manufacturing

For manufacturing the PDMS structure, at first a mold was designed (SolidWorks, Dassault Systemes, France) and then manufactured with the Replicator+ 3D printer (MakerBot, USA) using a polylactide acid (PLA) filament. The PDMS solution (Sylgard 184, Dow Corning, USA) was prepared in standard 10:1 proportion of pre-polymer and curing agent. The mold was then placed in the center of a μ-Dish chamber (ibidi GmbH, Germany), where the PDMS was poured, and degassing was performed in a vacuum jar at room temperature for 45 min. In parallel, carbon rods (Sigma-Aldrich, Germany) were cut at a length of 26 mm. Once the PDMS structure was polymerized at 50°C for 24h, in order to electrically connecting the carbon rods to ELETTRA, a hole was drilled through each carbon rod and, with the help of a cannula, two platinum wires (Polyfil AG) were passed through the PDMS structure and then connected to each carbon rod. The carbon rods were then inserted into the PDMS structure to serve as electrodes. Platinum wires, which are thin enough to pass through the space between the μ-Dish chamber and the lid, were cut long enough to access the external environment guaranteeing sterility to the cultured cells.

Lumped-parameter model assumptions

The three parameters of the Randles Cell (R_e, R_p, C_p) were evaluated considering the materials and geometry of the chamber described in section 2.2.

To determine R_e it must be taken into account the conductivity of the solution σ and the geometry of the electrolyte where the current flows in. For an electrode area *A* exposed to the electrolyte carrying a uniform current, being *d* the spacing between the electrodes, the solution resistance is calculated as follows (Tandon et al., 2008):

$$R_e = \frac{d}{\sigma A}$$

where *σ* is the conductivity of the solution and *A* is the area of the electrode exposed to the solution.

The value of A was calculated assuming that the cylindrical carbon rod electrodes in the chamber expose 2/3 of their lateral surface to the electrolyte:

$$A = 2\pi \cdot r \cdot l \cdot \frac{2}{3}$$

Considering the length of the portion exposed by the electrode to the electrolyte l = 20 mm, the electrode radius *r* = 1.5 mm a the interelectrodic distance *d* = 1 cm, the area resulted *A* = 1.26 cm^2.

Considering the value for the conductivity of the culture media reported in literature (1.5 S/m (Tandon et al., 2011)), R_e was estimated at 53 Ω.

The polarization resistance R_p and the double layer capacitance C_p were evaluated starting from literature data relative to electrochemical impedance spectroscopy (EIS) studies which gave the normalized values over a unit area (R_p/A= 4.06*10^{13} Ω/cm^2; C_p/A= 190 µF/cm^2 (Tandon et al., 2006)) and resulted R_p = 5.13 x 10^8 MΩ and C_p = 240 µF.

Tandon, N., Cannizzaro, C., Figallo, E., Voldman, J., and Vunjak-Novakovic, G. (2006). Characterization of Electrical Stimulation Electrodes for Cardiac Tissue Engineering. in *2006 International Conference of the IEEE Engineering in Medicine and Biology Society* (New York, NY: IEEE), 845–848. doi: 10.1109/IEMBS.2006.259747.

Tandon, N., Marsano, A., Cannizzaro, C., Voldman, J., and Vunjak-Novakovic, G. (2008). Design of electrical stimulation bioreactors for cardiac tissue engineering. in *2008 30th Annual International Conference of the IEEE Engineering in Medicine and Biology Society* (Vancouver, BC: IEEE), 3594–3597. doi: 10.1109/IEMBS.2008.4649983.

Tandon, N., Marsano, A., Maidhof, R., Wan, L., Park, H., and Vunjak-Novakovic, G. (2011). Optimization of electrical stimulation parameters for cardiac tissue engineering. *J. Tissue Eng. Regen. Med.* 5, e115–e125. doi: 10.1002/term.377.

Movie analysis

From the recorded movies of the electrically paced samples, cardiomyocyte contractility was assessed by measuring the peak amplitude (PA) of the contractions, defined as the maximum displacement of each CM during a contraction, and the contraction time delay (CTD), defined as the maximum time delay between the contractions of different CMs following a single pacing pulse.

For this purpose, the movies, acquired using a 10X objective lens at 30 fps with the live-imaging microscope incubator (ZEISS X91, Olympus, Japan), were analyzed with TrackMate, a Fiji software (NIH, USA) tracking plugin, and processed with a custom Matlab code. Briefly, the movies in Fiji were divided in frames, each frame corresponded to 0.033 s of movies. In TrackMate, the estimated object diameter was set at 15 µm and the quality threshold at 50, using the LoG detector. The program tracked the movement of points through the different frames and generated their trajectory using the Simple LAP tracker. The trajectories were eliminated if they had gaps of tracking, or their displacements were greater than 10 µm between two consecutive frames, thus false trajectories were excluded. Ten trajectories per sample were randomly selected manually, to ensure that the trajectory corresponded exactly to the movement of the cells. For each trajectory, 4 cell contractions were recorded. The X and Y coordinates of the trajectories were exported to an Excel file and the signals were processed with a custom Matlab code. For each trajectory, the displacement magnitude was obtained considering as reference the X and Y coordinates of the points of an instant in which the cells were relaxed.

To calculate the PA, for each trajectory the local maxima of the displacement magnitude were extracted and then averaged, obtaining 10 PA values for each movie (Supplementary Figure S4).

To calculate the CTD, for each paced contraction, the time intervals between the trajectories' peaks were calculated and the maximum time interval value was considered as the CTD, obtaining 4 CTD values for each movie (Supplementary Figure S4). Values were grouped for each experimental condition and expressed as mean ± SD.

Supplementary Table S1. Electrical conductivity and relative permittivity values of the modelled sub-domains.

	PDMS	Carbon Rods	Medium	PE derivate	Air
**Electrical conductivity (S/m)**	0.83×10^{-12} (Pavesi et al., 2014)	$1{,}28 \times 10^{6}$	1.5 (Tandon et al., 2011)	1×10^{-15}	0
**Relative permittivity**	2.69 (Tsai et al., 2017)	12	80 (Chen et al., 2009)	2.3	1

Chen, M.-T., Jiang, C., Vernier, P. T., Wu, Y.-H., and Gundersen, M. A. (2009). Two-dimensional nanosecond electric field mapping based on cell electropermeabilization. *PMC Biophys.* 2, 9. doi: 10.1186/1757-5036-2-9.

Pavesi, A., Soncini, M., Zamperone, A., Pietronave, S., Medico, E., Redaelli, A., et al. (2014). Electrical conditioning of adipose-derived stem cells in a multi-chamber culture platform: Electrical Conditioning of Adipose-Derived Stem Cells. *Biotechnol. Bioeng.* 111, 1452–1463. doi: 10.1002/bit.25201.

Tandon, N., Marsano, A., Maidhof, R., Wan, L., Park, H., and Vunjak-Novakovic, G. (2011). Optimization of electrical stimulation parameters for cardiac tissue engineering. *J. Tissue Eng. Regen. Med.* 5, e115–e125. doi: 10.1002/term.377.

Tsai, P. J., Nayak, S., Ghosh, S., and Puri, I. K. (2017). Influence of particle arrangement on the permittivity of an elastomeric composite. *AIP Adv.* 7, 015003. doi: 10.1063/1.4973724.

Supplementary Table S2. Peak current flowing inside the culture chamber for all the tested stimulation conditions. To compare monophasic and biphasic stimulations, current values were expressed as absolute values.

**Stimulation voltage (V)**	**Peak current (mA)**					
	**Monophasic**			**Biphasic (symmetric)**		
	Output 1	Output 2	Output 3	Output 1	Output 2	Output 3
1	16.78 ± 0.19	16.70 ± 0.82	17.09 ± 0.78	21.01 ± 0.77	21.15 ± 0.87	20.75 ± 0.58
2	33.05 ± 0.19	32.65 ± 0.72	32.91 ± 0.67	39.65 ± 0.28	40.18 ± 0.31	40.22 ± 0.43
3	47.63 ± 0.23	47.24 ± 0.31	47.37 ± 0.68	57.24 ± 0.28	59.09 ± 0.37	58.56 ± 0.45
4	63.33 ± 0.41	62.72 ± 0.43	62.98 ± 1.11	76.15 ± 0.46	78.43 ± 0.37	77.73 ± 0.45
5	77.84 ± 0.46	77.40 ± 0.03	77.05 ± 0.45	94.00 ± 0.50	95.93 ± 0.28	95.50 ± 0.45
6	93.94 ± 0.37	93.50 ± 0.83	92.44 ± 0.28	112.72 ± 0.56	114.30 ± 0.83	112.89 ± 0.74
7	109.05 ± 0.02	108.70 ± 0.74	107.65 ± 0.74	130.12 ± 0.03	130.48 ± 0.74	129.60 ± 0.85
8	124.71 ± 0.56	123.83 ± 0.91	123.12 ± 0.03	147.71 ± 0.03	147.71 ± 0.05	145.60 ± 0.74
9	137.37 ± 0.56	138.95 ± 0.03	137.37 ± 0.56	162.31 ± 1.45	163.89 ± 0.74	161.43 ± 1.11
10	154.78 ± 0.04	153.91 ± 0.93	153.03 ± 0.03	179.72 ± 1.11	179.19 ± 0.99	175.68 ± 0.56
11	169.20 ± 0.74	167.27 ± 0.56	167.27 ± 0.56	195.20 ± 0.03	193.61 ± 0.56	191.15 ± 0.85
12	170.79 ± 0.56	167.27 ± 0.56	169.03 ± 0.56	205.80 ± 1.38	205.36 ± 0.03	205.36 ± 0.04

Supplementary Table S3. Technical specifications of ELETTRA and commercial electrical stimulators.

	**ELETTRA**	**Grass s88x**	**IonOptix CPace EM**
**Outputs**	3	2	1-8
**Waveform type**	Monophasic/biphasic with tunable half-waves	Monophasic/symmetric biphasic	Symmetric biphasic
**Voltage (V)**	Monophasic: 0.25-12<br>Biphasic: ± 0.25 - ± 12	Monophasic: 0.25-24<br>Biphasic: ± 0.25 - ± 12	± 0.1 - ± 40
**Frequency (Hz)**	0.5 - 10	0.01 - 1000	0.01 - 99
**Pulse duration**	1-10 ms	0.01 ms - 99 s	0.4-10 ms
**Peak stimulation current (mA)**	700	1000	240
**Length (cm) x width (cm) x height (cm)**	21 x 18 x 7	48.3 x 31.8 x 13.4	NA
**Weight (kg)**	0.7	4.5	3.9
**Cost**	€ 250 - 300	€ 1200 - 1500	€ 9000 - 15000

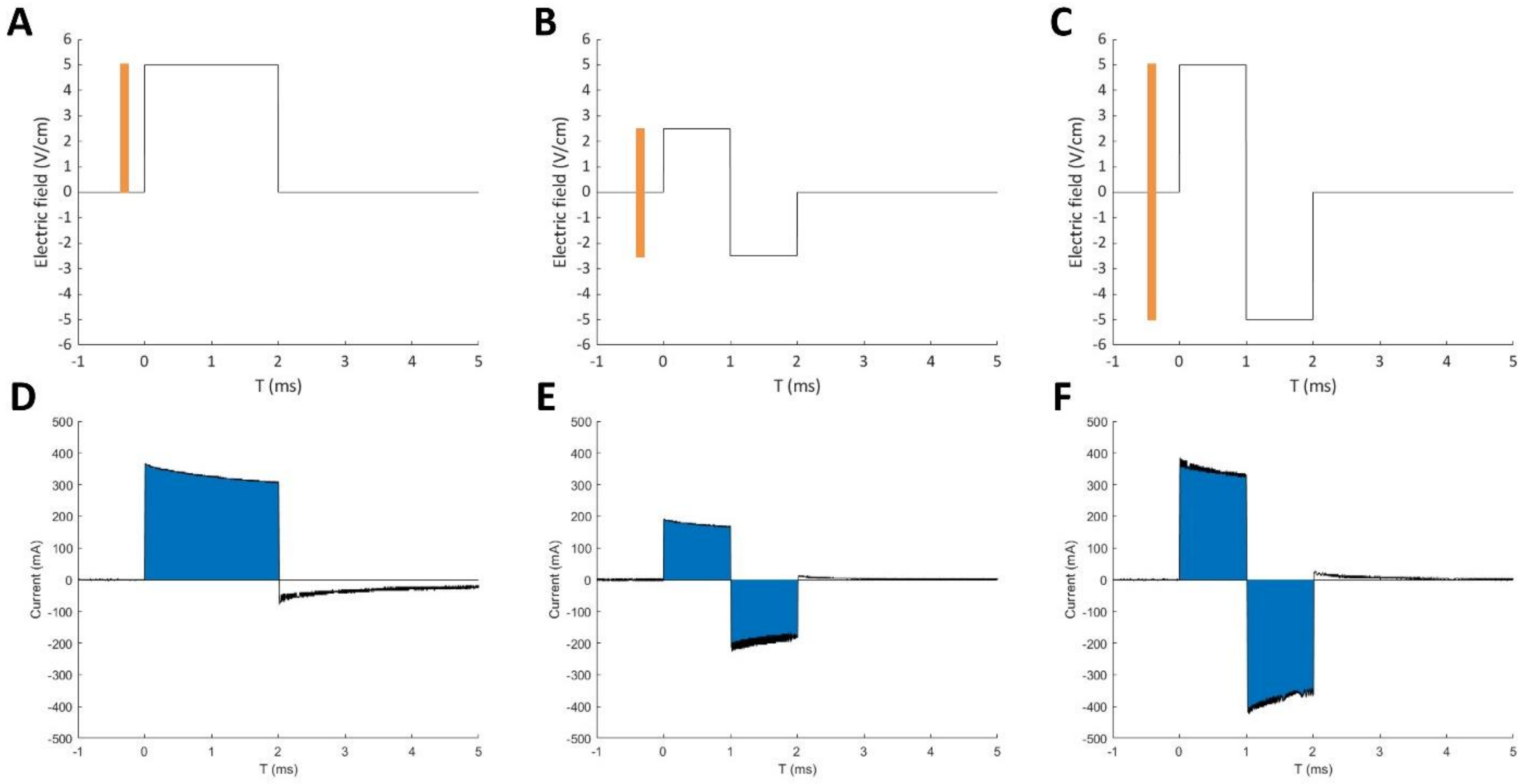

Supplementary Figure S1. Comparison of the ES modes used in biological experiments. The absolute value of the electric field variation (orange bar) of the monophasic ES at 5 V/cm (A) and of biphasic ES at ± 2.5 V/cm (B) are equal, while that of biphasic ES at ± 5 V/cm (C) is twice as much. The total charge (blue area) delivered by monophasic ES at 5 V/cm (blue area, D) is twice the charge delivered by biphasic ES at ± 2.5 V/cm (E) and equal to that of biphasic ES at ± 5 V/cm (F).

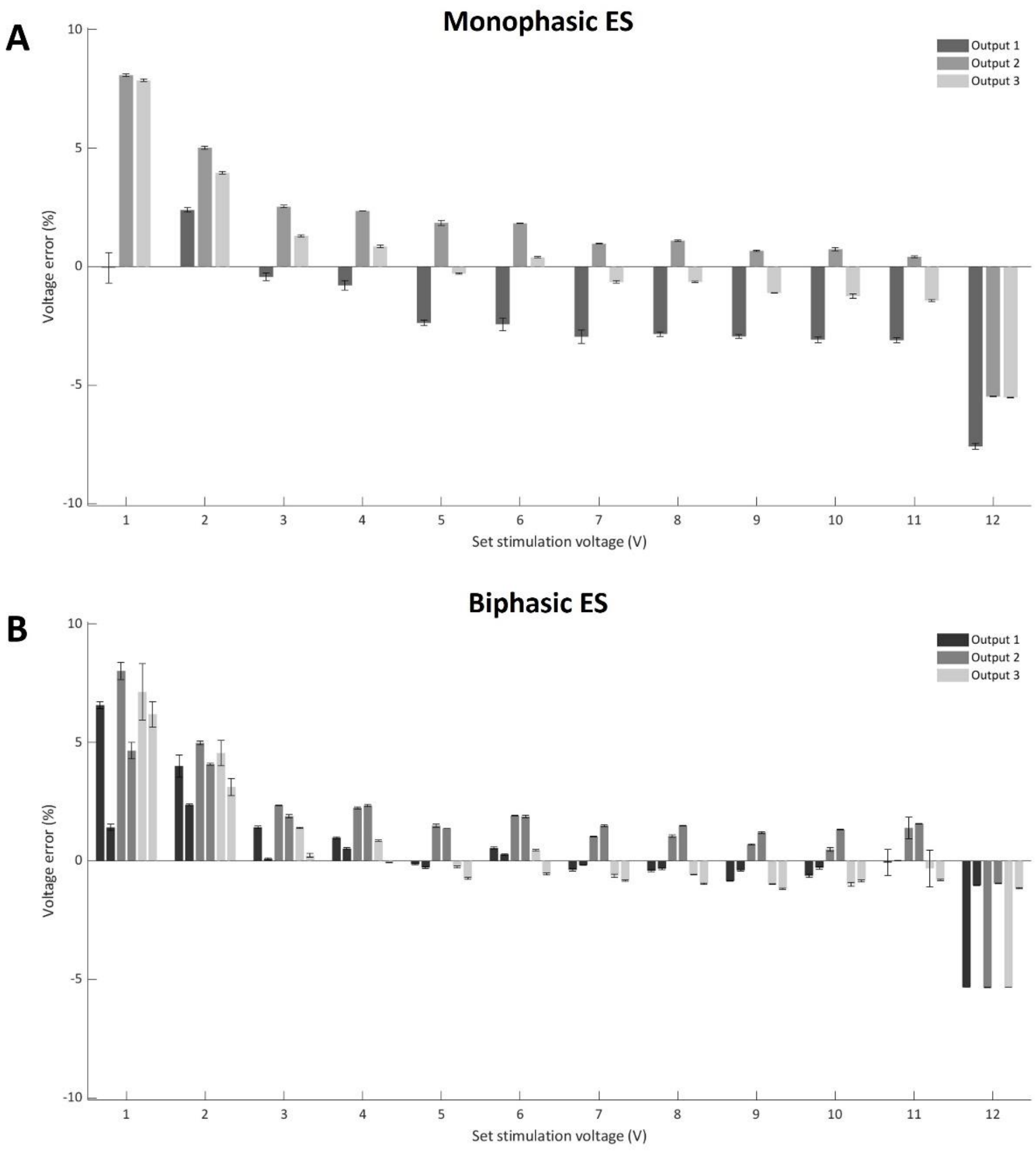

Supplementary Figure S2. Percentage errors of the measured voltage with respect to the imposed voltage for monophasic ES (A) and biphasic ES (B)

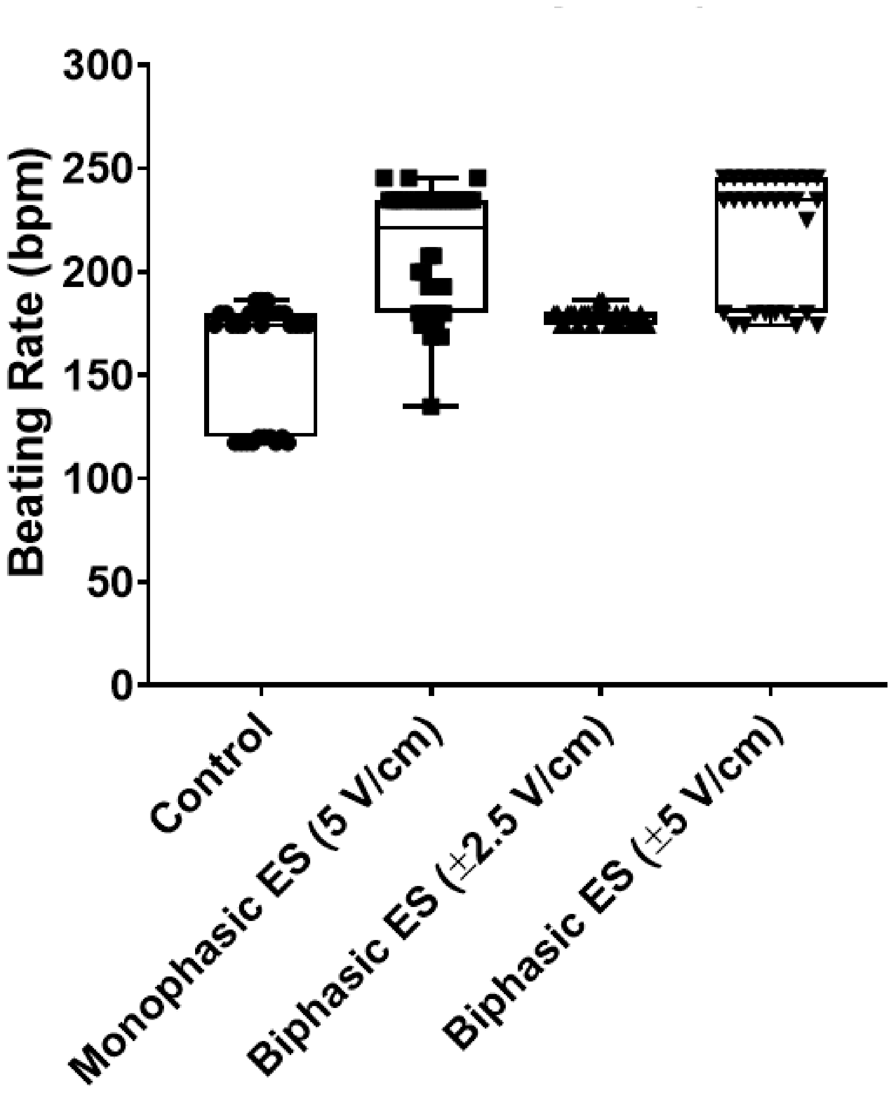

Supplementary Figure S3. Beating rate of contracting CMs during the MCR assessment for the different culture conditions: Control (no stimulation); Monophasic ES (5 V/cm, 1 Hz, 2 ms); Biphasic ES (±2.5 V/cm, 1 Hz, 2 ms); Biphasic ES (±5 V/cm, 1 Hz, 2 ms). For each condition, n = 4 replicates.

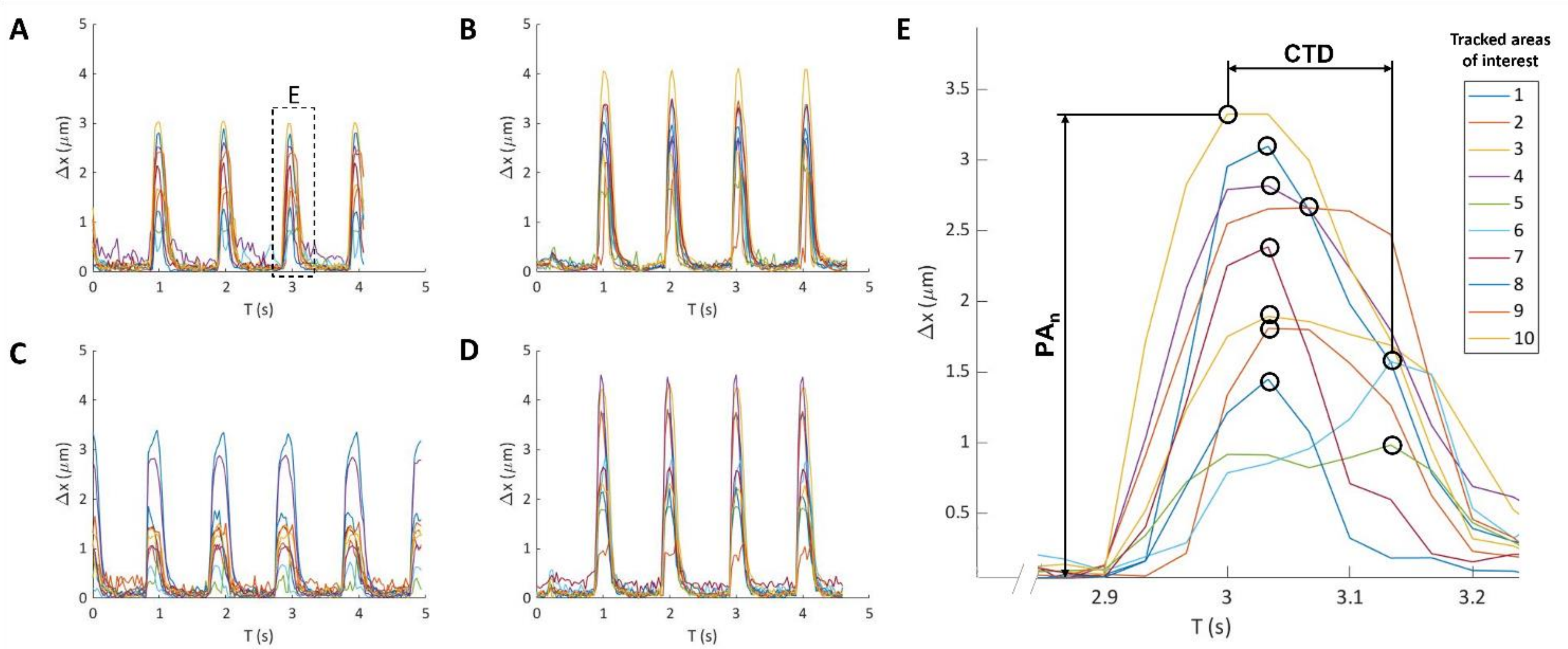

Supplementary Figure S4. Movie analysis for cardiomyocyte contractility assessment. Displacement magnitudes of consecutive contractions of CMs for the different culture conditions. (A) Control (no stimulation). (B) Monophasic ES (5V/cm, 1 Hz, 2 ms). (C) Biphasic ES (±2.5V/cm, 1 Hz, 2 ms). (D) Biphasic ES (±5V/cm, 1 Hz, 2 ms). (E) Detail of (A), where explanatory extracted values of peak amplitude (PA) and contraction time delay (CTD) are showed

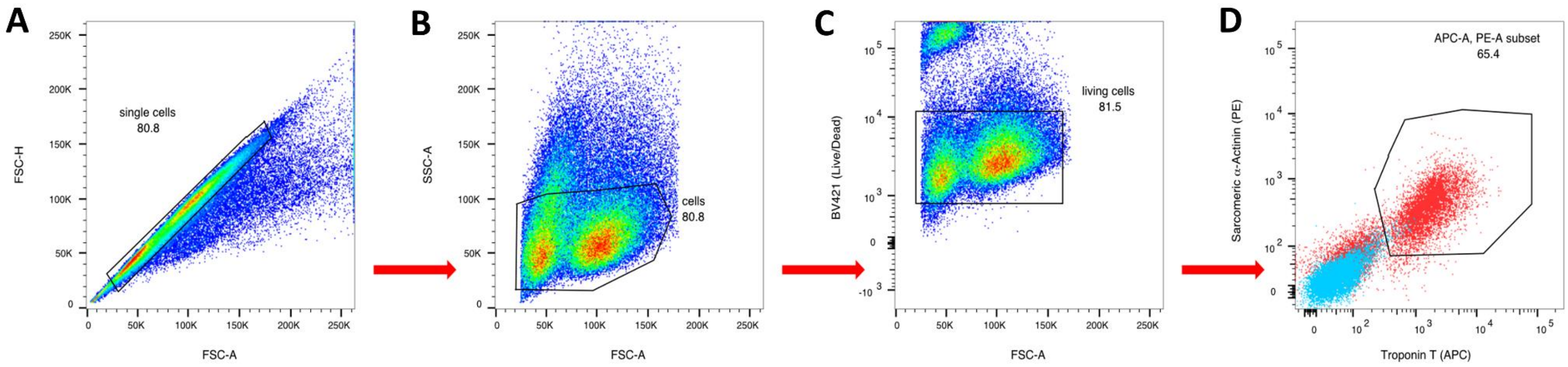

Supplementary Figure S5 Representative plots of the gating strategy for freshly isolated cardiac cells from neonatal rats (2-3 days old). Selection based on (A) single cells, (B) morphological parameters, (C) living cells. (D) Full stained sample for cardiomyocyte markers (Sarcomeric α-Actinin and Troponin T in red and unstained control in blue).

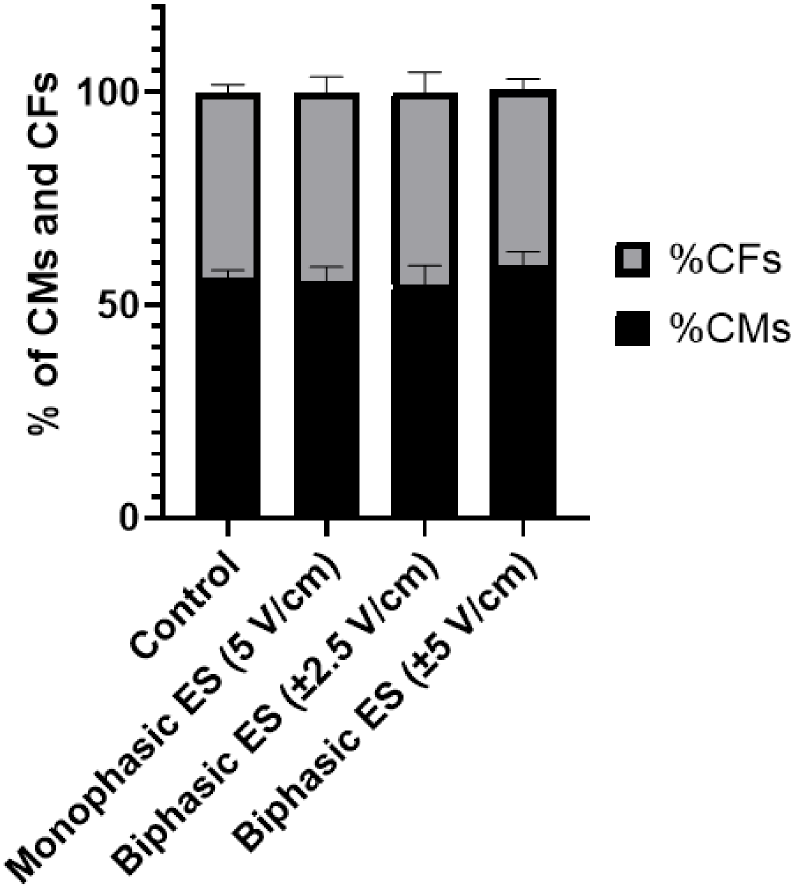

Supplementary Figure S6. Percentage of CMs and CFs after 7 days of cells cultured with (1) no stimulation, (2) monophasic ES (5V/cm, 1 Hz, 2 ms), (3) Biphasic ES (±2.5V/cm, 1 Hz, 2 ms) and (4) Biphasic ES (±5V/cm, 1 Hz, 2 ms) waveforms. For each condition, n = 4 replicates from 2 independent experiments.
